# Frataxin Traps Low Abundance Quaternary Structure to Stimulate Human Fe–S Cluster Biosynthesis

**DOI:** 10.1021/acs.biochem.4c00733

**Published:** 2025-02-05

**Authors:** Seth A. Cory, Cheng-Wei Lin, Shachin Patra, Steven M. Havens, Christopher D. Putnam, Mehdi Shirzadeh, David H. Russell, David P. Barondeau

**Affiliations:** † Department of Chemistry, Texas A&M University, College Station, Texas 77842, United States; ‡ Department of Medicine, University of California School of Medicine, La Jolla, California 92093-0660, United States

## Abstract

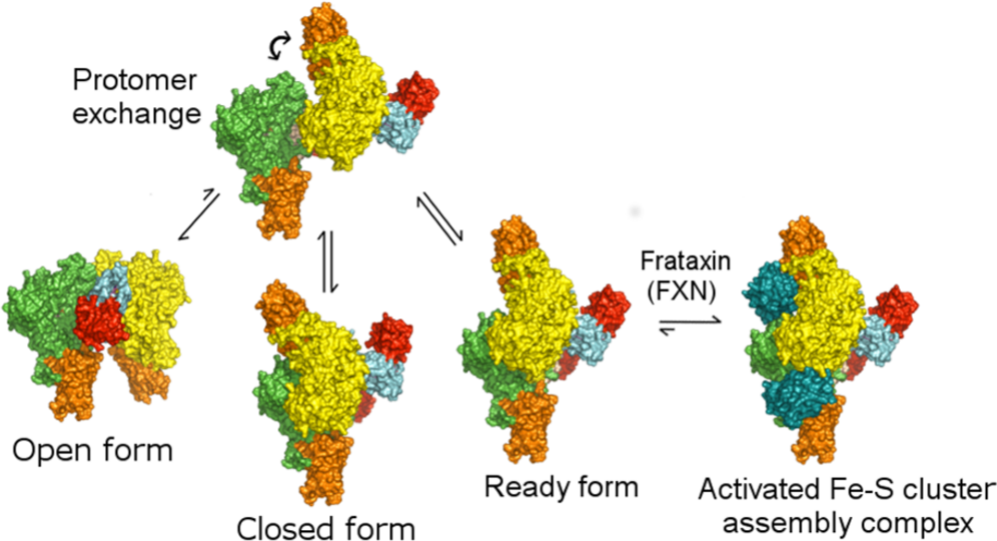

Iron–sulfur clusters are essential protein cofactors synthesized in human mitochondria by an NFS1-ISD11-ACP-ISCU2-FXN assembly complex. Surprisingly, researchers have discovered three distinct quaternary structures for cysteine desulfurase subcomplexes, which display similar interactions between NFS1-ISD11-ACP protomeric units but dramatically different dimeric interfaces between the protomers. Although the role of these different architectures is unclear, possible functions include regulating activity and promoting the biosynthesis of distinct sulfur-containing biomolecules. Here, crystallography, native ion-mobility mass spectrometry, and chromatography methods reveal the Fe–S assembly subcomplex exists as an equilibrium mixture of these different quaternary structures. Isotope labeling and native mass spectrometry experiments show that the NFS1-ISD11-ACP complexes disassemble into protomers, which can then undergo exchange reactions and dimerize to reform native complexes. Single crystals isolated in distinct architectures have the same activity profile and activation by the Friedreich’s ataxia (FRDA) protein frataxin (FXN) when rinsed and dissolved in assay buffer. These results suggest FXN functions as a “molecular lock” and shifts the equilibrium toward one of the architectures to stimulate the cysteine desulfurase activity and promote iron–sulfur cluster biosynthesis. An NFS1-designed variant similarly shifts the equilibrium and partially replaces FXN in activating the complex. We propose that eukaryotic cysteine desulfurases are unusual members of the morpheein class of enzymes that control their activity through their oligomeric state. Overall, the findings support architectural switching as a regulatory mechanism linked to FXN activation of the human Fe–S cluster biosynthetic complex and provide new opportunities for therapeutic interventions of the fatal neurodegenerative disease FRDA.

## Introduction

Iron–sulfur (Fe–S) clusters are essential inorganic cofactors found in proteins across all domains of life. These clusters play important roles in various biological processes, including oxidative respiration, DNA replication and repair, and catalytic transformations of substrates. The ISC biosynthetic pathway synthesizes Fe–S clusters in the mitochondria of eukaryotic cells and the cytosol for many prokaryotes.^1−3^ However, the substrates required for their synthesis, S^2–^ and Fe^2+^, contribute to oxidative stress by inhibiting respiratory complex IV and undergoing Fenton chemistry, respectively.^4,5^ As a result, multiple levels of post-translational regulation control eukaryotic Fe–S cluster biosynthesis, and defects in this biosynthetic pathway can lead to disease.^6^ These poorly understood regulatory mechanisms include the allosteric activator protein frataxin (FXN),^7−12^ the metabolite sensing acyl-carrier protein (ACP),^13−16^ and amino acid post-translational modifications.^17−19^ Understanding the details of these mechanisms is crucial for a comprehensive understanding of Fe–S cluster biosynthesis and may provide valuable insights into therapeutic interventions for human diseases.

A multiprotein assembly complex located in the mitochondrial matrix is responsible for synthesizing Fe–S clusters. The sulfur-hub of the assembly system exists as a stable subcomplex consisting of the pyridoxal 5′-phosphate (PLP) dependent cysteine desulfurase (NFS1),^20,21^ a member of the eukaryotic-specific LYRM superfamily (ISD11),^22−24^ and ACP.^13,15,16^ This subcomplex generates persulfide intermediates with subsequent transfer of the sulfane sulfur atoms to the scaffold protein ISCU2, where they are combined with ferrous iron and 2 electrons, likely provided by a ferredoxin protein,^25,26^ to synthesize [2Fe–2S]^2+^ cluster intermediates.^27^ The cysteine desulfurase complex is also involved in other critical cellular processes, such as sulfur trafficking for molybdenum cofactor biosynthesis and tRNA modifications.^28−30^ FXN binds to the assembly complex and stimulates Fe–S cluster biosynthesis.^7−12,25,27,31−33^ Notably, the loss of FXN is linked to developing the neurodegenerative disease Friedreich’s ataxia.^34^ Despite the considerable progress made in understanding the individual chemical steps accelerated by FXN, it is imperative that we conduct further research to fully comprehend the structural basis and physiological purpose of this regulation.

**Figure 1:**
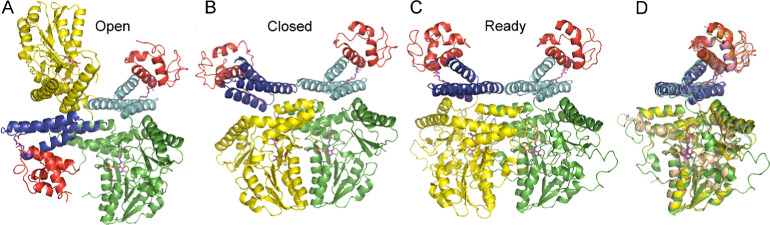
Comparison of different SDA_ec_ architectures. Structure of the SDA_ec_ complex in the (A) open (pdb: 5USR), (B) closed (pdb: 5WGB), and (C) ready (pdb: 6NZU; ISCU2 and FXN not shown) forms. NFS1 is shown in yellow and green, ISD11 in blue and cyan, and ACP_ec_ in red. Protein cofactors are shown in magenta. The differences in protomer–protomer interactions for the open, closed, and ready forms are largely due to rotation of the yellow/blue/red/protomers about the *y*-axis relative to the green/cyan/red protomers, which are shown in the same orientation in this figure. (D) Overlay of the subunits from one protomer for NFS1 (green, yellow, and wheat), ISD11 (cyan, blue, and purple), and ACP (red, orange, and pink) of the ready, closed, and open forms, respectively.

Structural studies have identified three different quaternary structures for the eukaryotic cysteine desulfurase complex, which is composed of NFS1-ISD11 associated with *Escherichia coli*ACP. This complex will be referred to as SDA_ec_ in this report. The first X-ray crystal structure exhibited an “open” architecture (Figure 1A),^15^ which differed dramatically from the prokaryotic homologue IscS. This open form exhibited an α_2_β_2_γ_2_ quaternary structure, where ISD11 molecules played a crucial role in mediating interactions between two NFS1-ISD11-ACP (αβγ) protomers. The open architecture has few direct interactions between the NFS1 subunits, unlike the extensive subunit interactions observed in IscS (Figure S1).^35^ A subsequent crystal structure revealed the SDA_ec_ complex can generate a second, distinct α_2_β_2_γ_2_ quaternary structure using an NFS1-NFS1 instead of an ISD11–ISD11 interface (Figure 1B).^16^ This “closed” SDA_ec_ architecture was also found to differ from the IscS–IscS dimer interface. When aligned, the twofold axes show a 10° rotation of each NFS1 subunit in the closed SDA_ec_ dimer compared to its IscS counterpart. Further structural studies revealed that the SDA_ec_ complex can form a third “ready” architecture (Figure 1C) upon binding of ISCU2 (SDA_ec_U)^16^ or both ISCU2 and FXN (SDA_ec_UF).^36^ The ready architecture uses a similar interface between NFS1 subunits as is observed in the IscS dimer (Figure S1). While the NFS1-ISD11-ACP_ec_ protomers are superimposable for the three forms (Figure 1D), they use different protein–protein interactions to generate the open, closed, and ready SDA_ec_ architectures.

The formation of different quaternary structures using distinct protein–protein interfaces is not a common occurrence, and the physiological function of these different assemblies remains a topic of active research. The similarity of the dimer interface between the ready SDA_ec_ architecture and IscS suggests that the ready form is the functional architecture for Fe–S cluster biosynthesis.^16,36−38^ However, the ready architecture does not provide a clear explanation for the essential functional requirement of ISD11, unlike the open architecture, which depends on ISD11 for protomer association (Figure 1A). Additionally, the ready architecture, which has similar active site and protein–protein interactions with IscS, does not easily explain the low activity and need for an activator in the eukaryotic Fe–S cluster assembly system. Moreover, the ready form does not account for additional differences from the prokaryotic system, such as the distinct binding characteristics of accessory proteins with their respective cysteine desulfurases and opposing activation/inhibition effects of FXN homologues.^15,25,31,39−42^ Interestingly, while small-angle X-ray scattering (SAXS) and cross-linking mass spectrometry studies provide evidence for the closed or ready form of the SDA_ec_ architecture in solution, other electron microscopy studies indicate that the open architecture is the predominant form for the SDA_ec_ complex.^15,16,43^

The eukaryotic Fe–S assembly complex has three architectures with distinct NFS1 active site conformations, reminiscent of the morpheein class of regulatory proteins. Morpheeins are known to control activity by shifting the equilibrium between different oligomeric forms that have distinct functionalities.^44^ However, there is no evidence that multiple cysteine desulfurase architectures exist in equilibrium or that the different forms have different activity profiles. Here, we employed a range of functional and biophysical approaches to examine the solution states of the SDA_ec_ complex. Our findings support an architectural switching model as a regulatory mechanism associated with FXN activation of the human Fe–S cluster biosynthetic complex. These results highlight the significance of understanding the conformational landscape of the SDA_ec_ complex, including the relationship between the open, closed, and ready forms and their roles in sulfur trafficking and the synthesis of sulfur-containing biomolecules.

## Results

### SDA_ec_ Preparation Method Does Not Affect Activity

We first investigated if different preparation methods for the SDA_ec_ complex might favor different architectures and influence the activity profile of the enzyme. Previously, researchers used slightly different expression and purification conditions to produce the SDA_ec_ complex, which they then used to crystallize the complex in open or closed forms.^15,16^ The open form was expressed in cells growing in an autoinduction media (herein named AI),^15^ whereas the closed form was induced in cells growing in a rich Terrific Broth media (herein named TB).^16^ The purification of the AI-prepared SDA_ec_ complex also includes additional purification steps. The SDA_ec_ samples prepared by these different methods did not significantly differ in catalytic properties when assayed under FXN-activated conditions (Figure S2). SDA_ec_ prepared under the AI conditions had a *k*_cat_ of 9.3 ± 0.5 min^–1^ and a *K*_M_ for cysteine of 22 ± 5 μM. When prepared under the TB conditions, SDA_ec_ had a *k*_cat_ of 11 ± 0.4 min^–1^ and a *K*_M_ for cysteine of 20 ± 3 μM. These kinetic constants were consistent with each other and with previous reports,^7,15,31^ suggesting that the preparation method does not substantially influence the activity profile of the SDA_ec_ sample.

### Small-Angle X-ray Scattering of SDA_ec_

**Figure 2:**
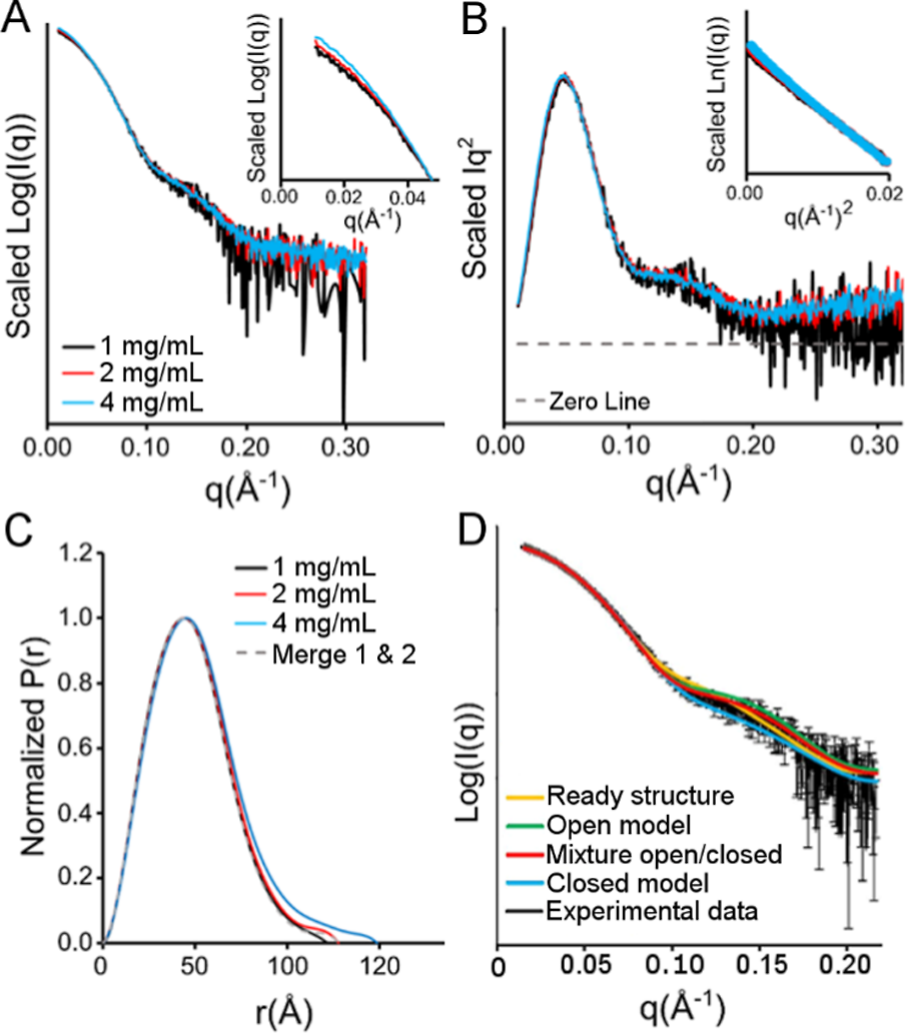
Small-angle X-ray scattering data for AI-prepared SDA_ec_. The SDA_ec_ complex was prepared using the AI method and examined under high salt conditions. (A) Overlay of buffer-subtracted scattering curves. Inset: concentration dependent aggregation revealed by overlay of low q region. Negative intensities are not shown. (B) Kratky plots for SDA_ec_ at multiple concentrations. Inset: concentration dependent aggregation shown by Guinier plot analysis. (C) Pair distribution functions for SDA_ec_ samples. (D) Fits to the experimental data for the calculated scattering curves from the ready SDA_ec_ structure (yellow; χ^2^ = 1.2), open model (green; χ^2^ = 2.1) and closed model (blue; χ^2^ = 2.1). The best two state model included the open (68%) and closed (32%) forms but did not significantly improve the fit (red; χ^2^ = 2.0).

We then investigated if the different SDA_ec_ preparation methods affected the solution conformation. SAXS curves of the AI-prepared SDA_ec_ sample (Figure 2) were collected, evaluated, and compared with previously analyzed SAXS samples generated with different preparation methods.^16,43^ We found that a high ionic strength buffer containing glycerol and TCEP maximized the stability of the complex and reduced concentration-dependent aggregation (Figure 2A). Kratky plots of the scattering indicated that the samples were folded (Figure 2B); however, we still observed minor concentration-dependent aggregation based on the behavior of the low q region of the scattering curve (Figure 2A, inset) and the Guinier analysis (Figure 2B, inset), as well as a concentration-dependent increase in *D*_max_ in the pair distribution function (Figure 2C). Due to these observations, we analyzed the lowest concentration sample, which provided a smooth pair distribution function, a *D*_max_ approximately the diameter of all three SDA_ec_ architectures (100–110 Å), and a calculated^45^ molecular weight that matched the expected molecular mass of 134 kDa (Table S1). Calculated scattering curves from the ready architecture, models of the open and closed forms, and mixtures of the different structures fit the experimental data similarly (Figure 2D). Overall, fitting calculated scattering curves or SAXS ab initio reconstructions from these and additional models that included limited molecular dynamic simulations failed to be sufficiently deterministic to assign an architecture for the AI-prepared SDA_ec_.

To compare our SAXS results with previous data from samples prepared by other groups, we reprocessed the scattering curves published by the Markley group,^43^ obtained from SASBDB,^46^ and the Cygler/Lill groups,^16^ which they kindly provided. The data collected by the Markley group^43^ closely resembled the data for our AI-prepared SDA_ec_ complex (Figure S3A); the *R*_g_ from Guinier analysis was 36.3 and 36.9 Å (Table S1), respectively. The data collected by the Cygler/Lill groups^16^ exhibited some concentration-dependent aggregation in the low q region (Figure S3B). Our reanalysis of the Cygler/Lill data (Table S2) is consistent with their reported *R*_g_ of 54.7 Å and *D*_max_ of approximately 180 Å.^16^ When we collected SAXS data with a lower ionic strength buffer comparable to that used by the Cygler/Lill groups, we obtained similar scattering curves for our AI-prepared SDA_ec_ sample, an *R*_g_ of 51.5 Å and a *D*_max_ of approximately 189 Å (Figure S3C and Table S2). Overall, the matching activity profiles and SAXS curves (Figure S3, Table S1, and Table S2)^16,43^ suggest that SDA_ec_ complexes prepared by the different groups have similar structure–function and solution properties.

### Crystallization of Different SDA_ec_ Preparations in Both Open and Closed Forms

**Figure 3:**
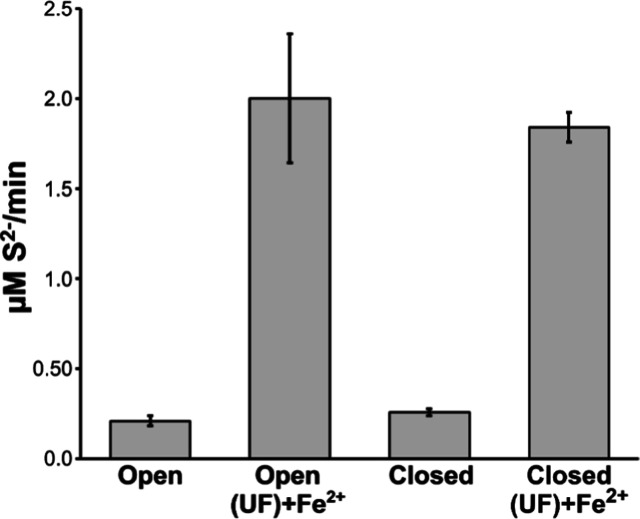
Isolated crystals in the open and closed forms exhibit similar cysteine desulfurase activities. Single crystals of the open and closed architectures were separately isolated, rinsed, and the resulting crystal slurries were dissolved by incubating with assay buffer at 37 °C for 15 min. The cysteine desulfurase activities of the open and closed samples were evaluated in the presence and absence of ISCU2, FXN, and Fe^2+^. Error bars are replicate errors (*n* = 6).

X-ray crystallography has provided the most substantial evidence of different SDA_ec_ architectures. We, therefore, investigated if the AI-prepared SDA_ec_ samples, used to generate crystals of the open architecture,^15^ and TB-prepared SDA_ec_ samples, used to generate crystals of the closed architecture,^16^ could generate both crystal forms. We buffer exchanged SDA_ec_ samples generated by the two methods and determined that each could be crystallized in the conditions for both the open and closed architectures (Figure S4). We further verified the presence of both crystal forms by screening the crystals on an X-ray diffractometer. After indexing the screened images (Table S3), it was clear that regardless of the preparation method, the SDA_ec_ complex could be crystallized into forms corresponding to both the open and closed architectures, indicating that both architectures exist in solution or that the two architectures can interchange. Next, we tested the activity of samples generated from the crystals of the open and closed forms as a mechanism to “freeze out” the individual architectures. We selected single crystals of each architecture, washed them to remove residual protein, and dissolved them in an assay buffer to measure activity with and without the activator subunit FXN. Interestingly, samples generated from both open and closed crystals show the characteristic order of magnitude activation by FXN (Figure 3). Together, these data indicate that the SDA_ec_ complex can exchange between open and closed forms or that both exist in solution. Moreover, these data suggest that adding FXN either activates both forms equally or, more likely, activates a single architecture generated by a quaternary structure rearrangement.

### SDA_ec_ Complex can Disassemble into Protomers and Undergo Exchange Reactions

**Figure 4:**
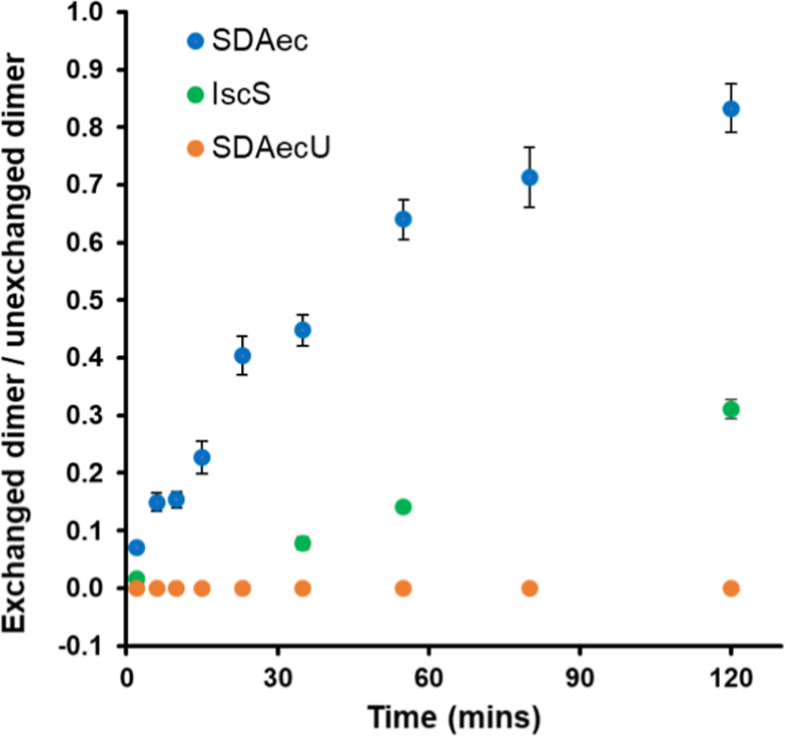
Protomer exchange for cysteine desulfurase complexes. Kinetics of an exchange reaction monitored by native mass spectrometry using a 1:1 ratio of His-tagged ^14^N-SDA_ec_ (^14^N^14^N) and ^15^N-SDA_ec_ (^15^N^15^N) complexes (blue). The *Y* axis is shown as the ratio of the amount of exchanged dimer (^15^N^14^N) divided by the sum of unexchanged dimer (^14^N^14^N and ^15^N^15^N). His-tagged and untagged versions of IscS undergo a similar exchange reaction monitored by native MS (green). Preincubation of ^14^N-SDA_ec_ and ^15^N-SDA_ec_ samples with ISCU2 completely inhibited the subsequent exchange reaction (orange). Error bars are replicate errors (*n* = 3).

The ability of SDA_ec_ samples to crystallize into both open and closed architectures led us to hypothesize that the distinct SDA_ec_ α_2_β_2_γ_2_ quaternary structures are in equilibrium. Based on the structures of the different architectures, interchange could occur via dissociation and reassociation of αβγ protomers or individual subunits. To test this hypothesis, we separately generated SDA_ec_ uniformly labeled with either ^15^N or ^14^N, mixed the two samples, and used native mass spectrometry to monitor if these complexes underwent subunit exchange reactions. Upon combining equimolar amounts of ^15^N- and ^14^N-labeled SDA_ec_, an intermediate-mass species consistent with the exchange of entire αβγ protomers to generate a ^15^N-SDA_ec_–^14^N-SDA_ec_ mixed complex was observed (Figure S5). In contrast, we did not observe masses suggesting the exchange of individual subunits. The protomer exchange for SDA_ec_ reached an exchanged-to-unexchanged ratio of 0.83 at 120 min (Figure 4 and Table S4); the theoretical maximum for this ratio is 1.0, corresponding to a completely exchanged equimolar mixture. These results reveal that the SDA_ec_ α_2_β_2_γ_2_ complexes can dissociate and reassemble αβγ protomers and suggests a model for switching between the open, closed, and ready architectures.

Native MS analysis of SDA_ec_ revealed primarily dimeric complexes with no significant population of protomeric species (Figure S6), consistent with the equilibrium favoring the α_2_β_2_γ_2_ complex. A small amount of tetrameric complex was also observed, which might explain the concentration-dependent aggregation observed in the SAXS experiments. Notably, for the protomer exchange reaction to occur, both the ^15^N-SDA_ec_ and ^14^N-SDA_ec_ complexes must dissociate and generate protomers at the same time, which would seemingly be a rare event and explain the modest protomer exchange kinetics. Similar native MS control experiments using the *E. coli* cysteine desulfurase IscS dimer with or without a His-tag revealed an even slower exchange process (reaching an exchanged-to-unexchanged ratio of 0.31 at 120 min). Preincubating saturating amounts of ISCU2 or ISCU2 plus FXN with the SDA_ec_ complex or IscU with IscS inhibited these exchange reactions (Figure 4 and Table S4), suggesting that they further shift the equilibrium away from protomeric forms. Next, we tested whether the presence of the His-tag influenced the SDA_ec_ protomer exchange reaction or the equilibrium between open, closed, and ready architectures. The protomer exchange reaction for SDA_ec_ lacking the His-tag on the NFS1 N-terminus was slower than the tagged material and required 24 h to reach an exchanged-to-unexchanged ratio of 0.79 (Table S4). This result is consistent with the his-tag influencing either the dissociation of the SDA_ec_ complex to form αβγ protomers or the reassembly of α_2_β_2_γ_2_ complexes. To examine whether changes in the exchange reaction affected activity, we tested the ability of untagged SDA_ec_ to be activated by FXN in the presence of 1 mM l-cysteine and Fe^2+^ and found similar activation (unactivated = 1.30 ± 0.01 μM S^2–^/min·μM NFS1; activated = 7.88 ± 0.34 μM S^2–^/min·μM NFS1) to the tagged SDA_ec_.^15^ These results indicate that although the native MS exchange assay provides evidence for SDA_ec_ protomer formation and reassembly of the native complex, the exchange kinetics do not correspond to the FXN activation phenomena (see Discussion).

### Interconvertible Forms of the SDA_ec_ Complex in Solution

**Figure 5:**
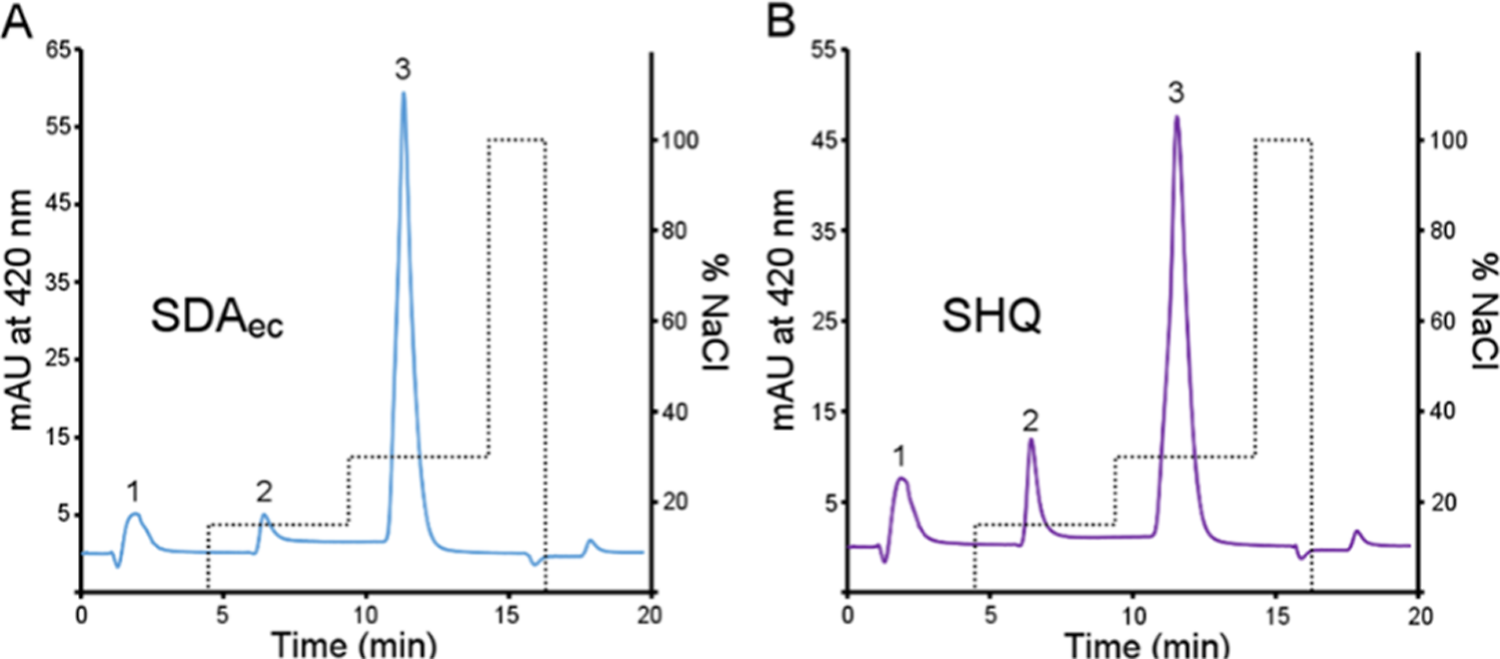
Separation of different SDA_ec_ forms by cation exchange chromatography. Different species were eluted for cysteine desulfurase samples from a cation exchange column using a step salt gradient. The PLP cofactor for SDA_ec_ samples was monitored at 420 nm. (A) The native SDA_ec_ sample (30 μM) had a major species that eluted at ∼12 min (peak 3) and a minor species that eluted at ∼7 min (peak 2). (B) The SHQ (30 μM) variant showed a larger initial population of peak 2 than SDA_ec_. Note that peak 1 likely results from the premature elution of the species in peak 2 due to the inability to remove all the salt from the injected sample for stability purposes.

We discovered that different forms of AI-prepared SDA_ec_ could be separated using a high-resolution cation exchange column. Native SDA_ec_ reproducibly separated into major (peak 3) and minor (peak 2) species (Figure 5A). We hypothesized that the species separated by cation exchange chromatography corresponded to different SDA_ec_ architectures. Based on the predominance of the open form in SDA_ec_ solutions,^15^ we tentatively assigned peak 3 as the open architecture and peak 2 as the closed and/or ready form, which have similar surface charge properties. Next, we evaluated whether these species could interconvert. The major species (peak 3) was isolated, concentrated, diluted with the loading buffer, and then reinjected onto the column. This sample’s elution profile included peaks 2 and 3 (Figures S7A,B), suggesting conversion from the major to the minor species. These results indicate that SDA_ec_ samples contain multiple forms in solution that can interconvert on a minute time scale.

We designed the Q64S, P299H, and L300Q NFS1 triple variant (herein designated as SHQ) to shift the population from the open to the ready architecture and support the tentative cation exchange peak assignments. We hypothesized that substitutions were incorporated into NFS1 during evolution, weakening the IscS-like dimeric interface and promoting dissociation and formation of the other eukaryotic cysteine desulfurase architectures. The three residues of the SHQ variant were selected as they are conserved but different in the prokaryotic and eukaryotic cysteine desulfurases of the ISC pathway. The introduced SHQ substitutions were expected to reduce steric clashes near the N-terminus and form new hydrogen bonds across the protein–protein interface of the ready form for the SDA_ec_ complex (Figures S8 and S9). The SHQ variant exhibited a similar FXN-stimulated cysteine desulfurase activity (8 μM S^2–^/min·μM NFS1) to the native SDA_ec_ complex (Figure S10). Notably, the SHQ variant had a threefold greater cysteine desulfurase activity than the native SDA_ec_ complex without FXN (Figure S10). This SHQ variant also exhibited an enhanced peak 2 intensity in cation exchange chromatography (Figure 5B), consistent with the tentative assignment of the ready form. The increase in activity for the SHQ variant suggests that the peak 2 species is the functional form of the complex. Overall, cation exchange chromatography revealed at least two SDA_ec_ species in solution that are interconvertible and appear to correlate with cysteine desulfurase activity.

### FXN Converts the SDA_ec_ and SDA_ec_U Complexes from an Extended to a Compact Conformation

**Figure 6:**
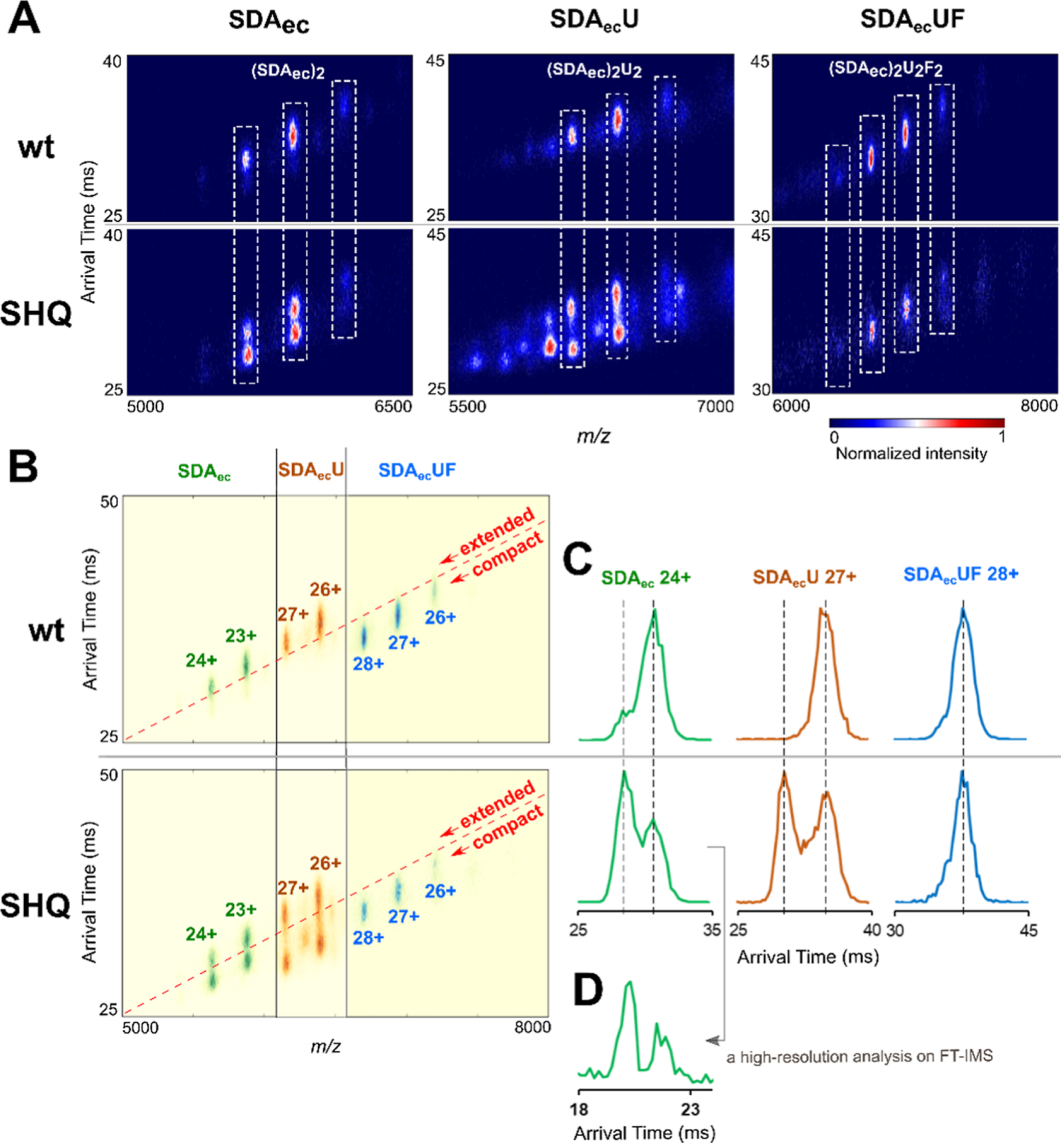
IM-MS analysis of SDA_ec_ samples reveals different forms. (A) IM-MS of native and variant SDA_ec_ as isolated complexes, in the presence of ISCU2, and with both ISCU2 plus FXN. Different charge states with appropriate *m*/*z* values are displayed for stoichiometric (inside the rectangles) and substoichiometric complexes (outside the rectangles). (B) Overlaid IM-MS spectra from (panel A). The SDA_ec_ and SDA_ec_U are predominantly in the slower migrating form (extended conformer trend line), whereas the S^SHQ^DA_ec_ and S^SHQ^DA_ec_U are enriched in the faster migrating species (compact conformer trend line). SDA_ec_UF and S^SHQ^DA_ec_UF exist as a single dominant species (compact conformer trend line). (C) Arrival time distribution of native and variant SDA_ec_ 24+, SDA_ec_U 27+, and SDA_ec_UF 28+. (D) Arrival time distribution of S^SHQ^DA_ec_ 24+ measured by the high-resolution FT-IMS instrument.

Next, we used native ion-mobility mass spectrometry (IM-MS) to investigate the conformational landscape and different architectures of the SDA_ec_ complex. IM-MS measures the arrival time of ions traveling through a drift tube filled with buffer gas molecules. Ions experience acceleration by an electric field and are slowed by collisions with gas molecules. Ions with a higher charge, a lower mass, or a compact shape travel faster through the drift cell. IM-MS charge state data for the SDA_ec_ complex revealed a large amount of a slower migrating (extended) form and a minor faster migrating (compact) species (Figure 6). There was minor variability in the amount of slower and faster migrating forms of SDA_ec_ depending on the batch and the presence of the his-tag (Figure S11A). Incubation of tagged and untagged SDA_ec_ samples at different temperatures before IM-MS analysis also slightly influenced the amount of extended and compact species (Figure S11B). Charge reduction analyses are consistent with the slower migrating complex being a more extended native-like conformation rather than a collisionally activated species (Figure S11C).

Next, we assessed whether the addition of ISCU2 and FXN affected the relative amounts of the extended and compact species in IM-MS. The SDA_ec_ complex exhibits approximately the same amount of extended and compact forms with or without ISCU2 (Figure 6), suggesting ISCU2 binds with similar affinity to the different forms. Strikingly, adding ISCU2 and FXN converts the SDA_ec_ complex to a single species following the faster-migrating trendline (Figure 6), consistent with FXN preferentially binding to the compact form. Theoretical calculations (Table S5) indicate that the ready and closed forms of the SDA_ec_ complex have similar collisional cross-sectional areas and are more compact than the open form.^47−52^ These calculations are consistent with the assignment of the slower migrating (extended) species as the open architecture and the faster migrating (compact) species as the closed and ready forms.

IM-MS analysis of the SHQ variant showed an enrichment of the faster migrating (compact) form compared to the SDA_ec_ complex (Figure 6). Remarkably, this is the same effect observed upon the addition of FXN and suggests that substitutions designed to stabilize the Ready architecture have a similar effect on SDA_ec_ quaternary structures as FXN binding. We propose a model (see Discussion) in which shifts in the equilibrium between architectures explain the functional activation of the eukaryotic cysteine desulfurase from either the SHQ substitutions or FXN binding. Overall, the results are consistent with the major peak in cation exchange and IM-MS experiments being a low-activity open architecture and the minor peak being a higher activity ready form. These results, in combination with the activity of samples generated from crystals from the different architectures, protomer exchange, and cation separation assays, suggest a dynamic interconversion between eukaryotic cysteine desulfurase architectures that appear to be a critical part of the FXN activation phenomenon.

## Discussion

Defining the physiological role and mechanistic details of FXN in the eukaryotic Fe–S assembly pathway has received significant attention due to its connection to Friedreich’s ataxia (FRDA).^34^ In 2010, in vitro assays revealed a role for FXN in stimulating the activity of the eukaryotic cysteine desulfurase complex.^7^ More recent studies show that FXN accelerates chemical steps associated with the mobile S-transfer loop, including the decay of the Cys-quinonoid PLP intermediate, the accumulation of a persulfide species on NFS1, and the sulfur transfer reaction to ISCU2.^11,12^ The analogous prokaryotic cysteine desulfurases, including *E. coli* IscS, do not require FXN-based activation and are functional without the additional subunits ISD11 and ACP. This suggests fundamental differences between eukaryotic and prokaryotic cysteine desulfurases.

The SDA_ec_ crystal structure in the open architecture provided the first evidence that these differences manifested as dramatic structural changes in the eukaryotic cysteine desulfurases.^15^ The open form features a solvent-exposed PLP, an incomplete substrate binding channel, and a quaternary structure that lacks significant NFS1–NFS1 interactions (Figure 1A), which are a hallmark of prokaryotic IscS cysteine desulfurases (Figure S1). A closed architecture crystal structure soon followed, revealing a significant NFS1–NFS1 interface^16^ but with different protein–protein interactions than IscS.^35,37,38^ Compared to IscS, the closed structure places the PLP cofactors 5 Å closer to one another and positions structural elements to potentially inhibit the function of the mobile loop cysteine in the sulfur transfer reaction (Figure S12). Remarkably, structures that included ISCU2 or ISCU2 and FXN revealed a third ready form of the SDA_ec_ complex^16,36^ with the same protein interface as IscS (Figure S1). The relationship between these different architectures, their connection to FXN activation, and their functional roles in sulfur transfer reactions remain incompletely understood.

**Figure 7:**
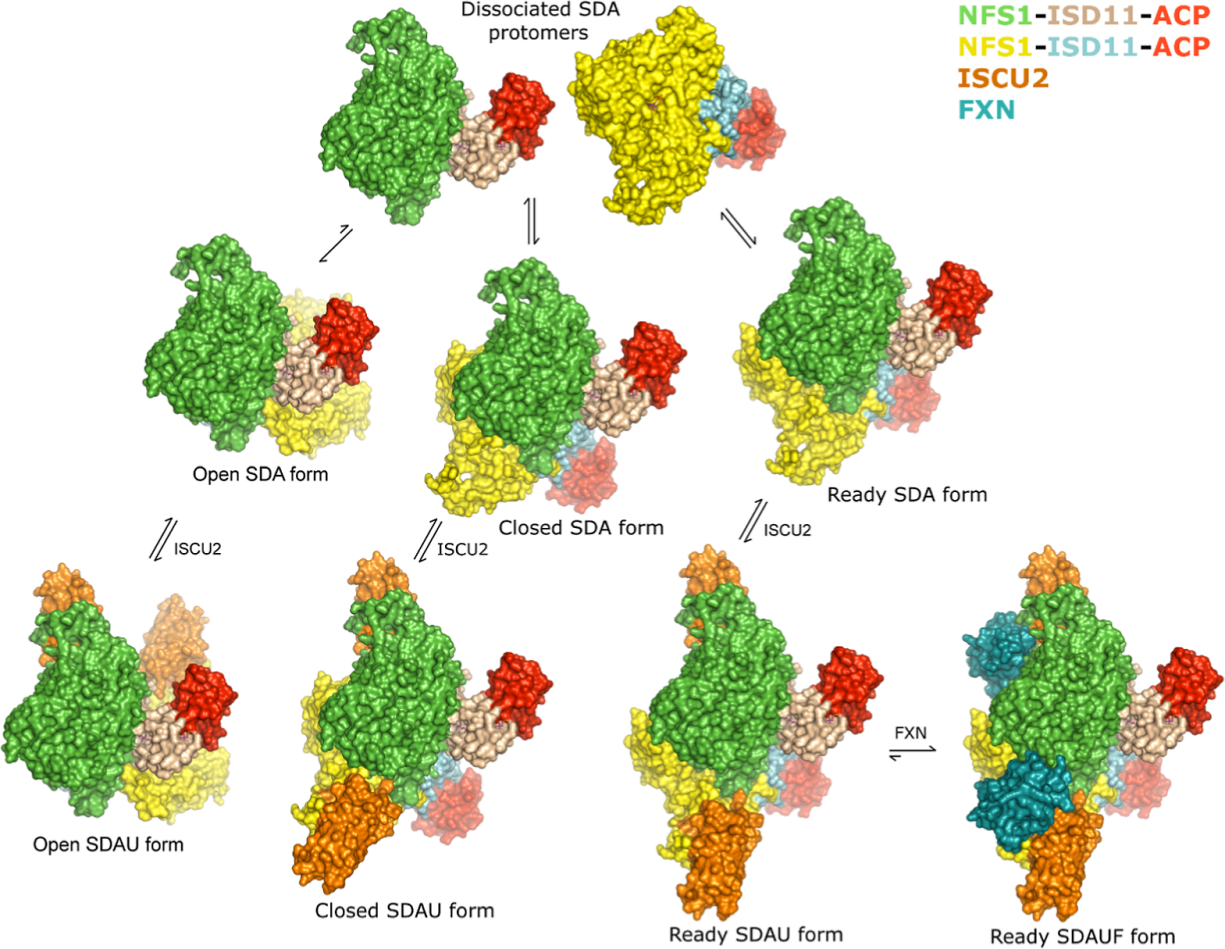
Morpheein model for the Fe–S cluster biosynthetic subcomplex. The NFS1-ISD11-ACP (SDA) complex exists as an equilibrium mixture of open (most abundant), closed, and ready architectures that are in equilibrium. ISCU2 binds to all three forms and does not significantly alter the equilibrium between forms. FXN binds to the SDAU ready form and locks the complex in the active conformation. NFS1 (green and yellow), ISD11 (wheat and cyan), ACP (red), ISCU2 (orange), and FXN (dark teal) subunits are shown as surfaces. The green NFS1 protomer is shown in the same orientation throughout the figure.

Here, we establish that the structure–function properties of SDA_ec_ samples are independent of the preparation method, that these samples consist of interconvertible equilibrium mixtures of different species, and that variant complexes or the binding of additional subunits can shift this equilibrium between states. Crystallographic studies reveal that SDA_ec_ samples exist as a mixture or can convert between the open and closed forms (Figure S4 and Table S3). IM-MS and cation exchange chromatography results also indicate multiple components in SDA_ec_ samples (Figures 5 and 6). We assigned the slower migrating species in IM-MS as the open form and the faster migrating species as the closed and/or ready form based on comparing experimental and calculated collisional cross-sectional areas (Table S5). This assignment is consistent with the enriched open-form population for SDA_ec_ samples in negative stain electron microscopy studies.^15^ IM-MS data also indicates that ISCU2 can bind to both extended and compact species of the SDA_ec_ complex and does not significantly shift the population between forms (Figure 6). Although there are no structural snapshots of the open or closed SDA_ec_ forms bound to ISCU2, the ISCU2 binding sites in the ready architecture^16,36^ are distant from the αβγ protomer interaction sites, suggesting that each architecture can bind ISCU2 (Figure 7).

Our results support and extend an architectural switch model in which FXN drives a change in the quaternary structure to activate the cysteine desulfurase and Fe–S cluster assembly activities (Figure 7).^11^ We provide evidence that SDA_ec_ samples exist as an equilibrium mixture of open, closed, and ready forms. Native mass spectrometry ^15^N–^14^N exchange assays show that SDA_ec_ samples can dissociate into αβγ protomers and reassemble into α_2_β_2_γ_2_ complexes (Figure 4). Such a complex-protomer-complex conversion process provides a possible route to interconverting between open, closed, and ready architectures. The αβγ protomers are not observed with direct biophysical techniques, indicating the protomer-complex equilibrium favors α_2_β_2_γ_2_ complex formation, and suggests the SDA_ec_ complex’s slow ^15^N–^14^N exchange kinetics may be due to low populations of ^15^N-αβγ and ^14^N-αβγ protomers, which need to coexist to produce a mixed isotope complex. It is unclear if ISCU2 must dissociate to form exchangeable SDA_ec_ species for the interconversion of SDA_ec_U architectures or if a similar complex-protomer-complex conversion occurs with αβγδ protomers. It is also unclear if the closed and ready forms can directly interconvert or if they must dissemble into protomers and reassemble. Our IM-MS data reveals that adding FXN converts the sample from existing as multiple species to one form, almost certainly the SDA_ec_UF observed in the cryo-EM structure.^36^ We view FXN as a “molecular lock” that preferentially binds to the ready form and stitches the two protomers together by simultaneously binding with both NFS1 subunits. In contrast, similar FXN interactions with both NFS1 subunits in the other architectures are impossible due to a steric overlap with the rotated protomers in the closed form and the new ISD11–ISD11 protomer interface in the open form (Figure S13). Driving the complex to the ready form would change the mobile S-transfer loop from a primarily disordered (open form) and potentially inhibited (closed form) to a functional trajectory (ready form) that promotes the PLP and sulfur transfer chemistry (Figure S12). The inability of the sulfur acceptor protein ISCU2, unlike FXN, to shift the population of these different architectures is consistent with its failure to activate the SDA_ec_ complex.^7^

This type of global structural rearrangement is uncommon. The closest system that describes this process is the morpheein model. Morpheeins are enzymatic systems that are in a dynamic equilibrium with a variety of different oligomeric or architectural states. The equilibrium between states is allosterically regulated, and for one oligomer or architecture to convert to the other, the system must dissociate, undergo a conformational change, and then reassociate.^53^ The architectural switch associated with FXN activation is much more rapid than the slow ^15^N–^14^N exchange kinetics because it only requires one complex to dissociate into a protomer, undergo a conformational change, and reassemble and not the simultaneous dissociation of two complexes followed by the formation of the mixed isotope dimer. Similarly, we propose conformational differences in protomers dictate the equilibrium population of the open, closed, and ready architectures. Stabilizing one of the quaternary structures over the others shifts the equilibrium and function of the complex (Figure 7). We tested this hypothesis by designing the SHQ variant, which showed enhanced activity and changes in IM-MS and cation exchange results consistent with a shifted population away from the open form and toward a closed or ready form. The equilibrium position also appears to be influenced by the sample incubation temperature (Figure S11). Small molecule effectors typically regulate human morpheein systems, and clinical mutations affect oligomeric distributions and activities for morpheein systems.^54−57^ In the human cysteine desulfurase system, a small molecule effector has not been identified, but FXN functions to alter the oligomeric distribution by locking the complex in the active form for Fe–S cluster biosynthesis. It will be interesting to evaluate whether clinical variants of NFS1, ISD11, ISCU2, and FXN^58−63^ alter the equilibrium between different architectures or potentially fail to lock the complex in the active form.

## Conclusions

These and previous studies provide substantial evidence that the eukaryotic cysteine desulfurase is a morpheein-like system that controls activity by its oligomeric form (Table S6). Assigning structure–function properties for the different architectures will require thoughtfully designed biochemical probes and high-resolution structural analysis. One possibility is that the open, closed, and ready architectures are part of a protein assembly based regulatory mechanism that controls sulfur transfer from the SDA_ec_ complex to acceptor proteins for Fe–S cluster assembly, molybdenum cofactor biosynthesis, and tRNA modifications. Consistent with this idea, we provide insights into the relationship between the eukaryotic cysteine desulfurase architectures and FXN activation for Fe–S cluster assembly. The ability of FXN to affect the equilibrium between architectures and trap a low abundance ready form to promote Fe–S cluster synthesis is consistent with previous biochemical and enzymology studies of FXN activation and supports the architectural shift model.^11^ Moreover, the ability of the SHQ variant to partially replace FXN function by shifting the population of quaternary structures suggests that molecules that drive a similar architectural switch may have potential applications as FRDA therapeutics.

## Materials and Methods

### Protein Preparation and Purification

#### Preparation of SDA_ec_

The NFS1(Δ1–55)-ISD11(S11A)-ACP_ec_ (SDA_ec_) was prepared following the published procedures describing the open^15^ and closed^16^ architectures. The two procedures used identical expression constructs that encode an N-terminal His_6_ tag on NFS1, which was not cleaved unless indicated. The two preparation methods differed in expression conditions, using autoinduction (AI conditions)^15^ or Terrific Broth (TB conditions)^16^ media, and slightly different purification procedures. A tobacco etch virus (TEV) protease cleavage site was introduced by mutagenesis into the original NFS1 plasmid to generate material with a cleavable His_6_ tag. The purification was conducted as described^15^ with a 4 °C overnight TEV cleavage step introduced after the cation exchange column to generate cleaved SDA_ec_. The digested product, which contained a single glycine residue before residue 56, was loaded onto a Ni-NTA column (5 mL; GE Healthcare) to remove the TEV protease. To generate ^15^N-labeled SDA_ec_, 2–6 L of N-5052 autoinduction media^64^ were inoculated with 8 mL of an overnight LB starter culture. The ^15^N-SDA_ec_ complex was purified as described,^15^ except that supplemental pyridoxal 5′-phosphate was not added during the preparation. The QuikChange protocol (Agilent) was used to introduce the Q64S L299H P300Q substitutions into the NFS1 plasmid (pet-15b).^15^ SDA_ec_ variants were purified using the same protocol as the native enzyme complex. The concentrations for the SDA_ec_ complexes were determined using an extinction coefficient of 10.9 mM^–1^ cm^–1^ at 420 nm.

#### Preparation of ISCU2 and FXN

A MEGAWHOP protocol^65^ was used to incorporate a TEV protease site and glutathione S-transferase (GST) into a pET-30a(+) vector containing ISCU2 (Δ1–35) and generate the C-terminally tagged construct *ISCU2-TEV-GST*. Further mutagenesis incorporated a C-terminal His_6_ tag to produce the *ISCU2-TEV-GST-His*_6_ construct. The *ISCU2-TEV-GST* plasmid was transformed into the *E. coli* strain BL21(DE3) for expression. Cells were grown at 37 °C to an OD_600_ of 0.5. Protein expression was induced with 0.1 mM isopropyl β-D-1-thiogalactopyranoside (IPTG) at 18 °C. Cells were grown overnight, harvested by centrifugation the following morning, and stored in a −80 °C freezer until use. The cell pellet from a 9 L culture was thawed and resuspended in GST buffer A (50 mM Hepes, 150 mM NaCl, pH = 7.8). Lysozyme (20 mg, Sigma-Aldrich) and protease inhibitor cocktail (20 mg, Sigma-Aldrich) were added to the suspension. The cells were lysed by two cycles of French press at 18,500 psi. Cell debris was cleared by centrifugation at 16,420 RCF for 30 min. The clarified lysate was loaded onto a manually packed GST-column (Prometheus) at 4 °C. Bound protein was eluted with GST buffer B (50 mM Hepes, 150 mM NaCl, 10 mM glutathione (GSH), pH = 7.8). The TEV digestion was conducted overnight at 4 °C and the products were loaded onto a Ni-NTA column (5 mL; GE Healthcare) to remove the TEV protease. The flow-through from the Ni-NTA column was concentrated to 20 mL, diluted to 150 mL with cation A buffer (50 mM Hepes, pH = 7.8), and loaded onto a cation exchange column (27 mL; POROS 50HS, Applied Biosystems) and eluted with a linear gradient of NaCl (0–1 M). The fractions containing ISCU2 were concentrated, brought into an anaerobic Mbraun glovebox (∼12 °C, <1 ppm of O_2_ as monitored by a Teledyne model 310 analyzer), and supplemented with 5 mM d,l-dithiothreitol (DTT) before loading onto a HiPrep 26/60 Sephacryl S100 HR column (GE Healthcare Life Sciences) equilibrated in size exclusion buffer (50 mM Hepes, 150 mM NaCl, pH = 7.5). The fractions corresponding to monomeric ISCU2 were collected, concentrated, and flash-frozen in liquid nitrogen for storage at −80 °C until use. For the *ISCU2-TEV-GST-His*_6_ construct, the same procedure was used, except that the cation exchange step was skipped. The preparation of FXN (Δ1–81) gene was previously described.^66^ Concentrations for ISCU2 and FXN were determined using extinction coefficients of 9970 and 26,930 M^–1^ cm^–1^ at 280 nm, respectively, as estimated by ExPASy ProtParam.^67^

#### Preparation of IscU and IscS

The *E. coli* proteins IscU and IscS were expressed and purified as previously described.^31^ The extinction coefficient of 6.6 mM^–1^ cm^–1^ at 388 nm was used to estimate the concentration of the PLP cofactor, which represented the concentration of active IscS, in 0.1 M NaOH. The extinction coefficient of 11,460 M^–1^ cm^–1^ at 280 nm was used to estimate the concentration of IscU.

### Activity Measurements of Purified Complexes

The cysteine desulfurase activity was determined using the methylene blue assay as described^15^ in assay buffer (50 mM Hepes, 250 mM NaCl, pH = 7.5). Reaction mixtures of 800 μL contained the following components: 0.5 μM SDA_ec_ (or the SHQ variant), 1.5 μM ISCU2, 1.5 μM FXN, 4 mM d,l-DTT, and 5 μM (NH_4_)_2_Fe(SO_4_)_2_ were incubated at 37 °C for 15 min before the addition of varying amounts of l-cysteine. Reactions were quenched after 6 min and the sulfide was quantified as described. The sulfide formation rate for each l-cysteine concentration was measured at least in triplicate. Data were fit using KaleidaGraph (Synergy Software) to a traditional Michaelis–Menten equation. The errors in the Michaelis–Menten parameters represent errors in the fit to the experimental data. FXN binding was evaluated as previously described.^60^

## Preparation of the SDA_ec_ Complex for Small-Angle X-ray Scattering

Purified AI-prepared SDA_ec_ was injected onto a Superdex 200 10/300 GL column (S200, GE Healthcare Life Sciences) equilibrated in 50 mM Hepes, 250 mM NaCl, pH = 7.2 to remove any aggregates from the freeze/thaw cycle of the sample. Yellow fractions were collected, pooled, and concentrated to approximately 10 mg/mL. Dialysis buttons (Hampton Research) were loaded with 50 μL of sample and sealed with a 3.5 kDa dialysis membrane disc (Hampton Research, Spectrum) washed thoroughly with Milli-Q H_2_O. Samples were then dialyzed into various buffers in 50 mL falcon tubes overnight at 4 °C before diluting within a 96-well plate. High salt conditions were defined as 100 mM sodium phosphate, 500 mM NaCl, 2% glycerol, 2 mM TCEP, pH = 8.0. Low salt conditions included 50 mM Hepes, 250 mM NaCl, 2% glycerol, 2 mM TCEP, pH = 7.5. The 96-well plate containing samples was sealed and shipped wrapped in ice packs to the SIBYLS beamline (12.3.1) at the advanced light source (ALS). The plate was stored at 4 °C and was centrifuged at 3700 rpm for 10 min before data collection. Data collection parameters can be found in Table S7.

### Small-Angle X-ray Scattering Data Collection and Analysis

Individual buffers and frames were analyzed for consistency. Buffers with the same composition and scattering profile were averaged using the ATSAS 2.8.4 package^68^ to generate an average buffer scattering curve. Sample frames were then individually subtracted from the averaged buffer in the RAW 1.5.1 package.^69,70^ Subtracted frames were then averaged in RAW at different time points to determine the onset of radiation damage. Exposure times, which included the least amount of radiation damage, were used for further analysis. The low q region was truncated based on Guinier analysis, and the high q region was truncated to 8/*R*_g_ before the pair distribution analysis. Additional information regarding Guinier analysis, pair-distribution function analysis, and curve fitting can be found in Tables S1 and S2. We used the same procedure to reanalyze the Markley and Cygler/Lill SAXS data, except that the scattering curve was truncated in the Guinier region due to significant aggregation^16^ or interference from the beam stop.^43^ Because the crystal structures of the open^15^ and closed^16^ SDA_ec_ architectures lacked a substantial number of non-hydrogen protein scatterers due to disordered regions in crystal structures (17.2% and 31.6%, respectively), we generated more complete models for calculating SAXS profiles by overlaying the NFS1-ISD11-ACP protomers (αβγ) of the cryo-EM ready form^36^ onto the open and closed architectures. The model for the ready architecture was generated by removing the ISCU2 and FXN subunits from the cryo-EM SDA_ec_UF structure.^36^

## Crystallization of SDA_ec_ from Different Preparation Methods

The open and closed forms of SDA_ec_ were crystallized as previously described^15,16^ using the AI-preparation and TB-preparation methods, respectively. A hanging-drop vapor diffusion method was used that included 500 μL of crystallization solutions in the well and a 4 μL drop (2 μL protein: 2 μL crystallization solution) on the coverslip. The AI-prepared SDA_ec_ in the closed form was prepared for crystallization by buffer exchanging the protein complex into 10 mM BIS-TRIS (pH 5.5), 200 mM NaCl, 20 mM KCl, 2 mM NaH_2_PO_4_, 2 mM Na_2_HPO_4_, 5% (v/v) glycerol, 1 mM d,l-DTT, and 75 mM imidazole by multiple rounds of concentration and dilution using a Vivaspin 500,100 kDa spin concentrator (GE Healthcare). The TB-prepared SDA_ec_ in the open form was prepared for crystallization by buffer exchanging the protein complex into 50 mM Hepes, 250 mM NaCl, 10% glycerol, pH = 7.5 or injected onto a Superdex 200 10/300 GL column (S200, GE Healthcare Life Sciences) equilibrated in 50 mM Hepes, 250 mM NaCl, 10% glycerol, pH = 7.5. The AI-prepared SDA_ec_ (177 μM) was crystallized in the open architecture at 22 °C with crystallization conditions generated by adding 5 mL of 40% acetone to 11.25 mL of 0.1 M CBTP (pH = 6.4), 0.3 M CsCl, 0.2 M d,l-allylglycine, 5 mM TCEP, and 8% PEG 3350. The AI-prepared SDA_ec_ (177 μM) without d,l-allylglycine was crystallized in the open architecture at 22 °C with crystallization conditions generated by adding 1.25 mL of 40% acetone to 11.25 mL of 0.1 M CBTP (pH = 6.4), 0.3 M CsCl, 5 mM TCEP, and 8% PEG 3350. The AI-prepared SDA_ec_ (220 μM) and the TB-prepared SDA_ec_ (226 μM) were crystallized in the closed architecture at 12 °C using a crystallization solution of 0.1 M MES (pH = 6.5), 0.3 M ammonium acetate, 0.02 M calcium acetate hydrate, 0.02 M calcium chloride dihydrate, and 15% isopropanol. The TB-prepared SDA_ec_ (177 μM) was crystallized in the open architecture at 22 °C using a crystallization solution of 0.1 M CBTP (pH = 6.4), 0.2 M CsCl, 0.2 M d,l-allylglycine, 5 mM TCEP, 10% PEG 3350, and 4% acetone.

## X-ray Data Collection, Indexing, and Unit Cell Determinations

Single crystals of SDA_ec_ in the open architecture were harvested and cryo-protected as previously described^15^ using a final concentration of 20% (v/v) PEG 400. Crystal trays of SDA_ec_ in the closed architecture were transferred to a 17 °C room where single crystals were harvested and cryo-protected as previously described.^16^ Diffraction data were collected using a rotating anode Cu K-α source and a Rigaku R-AXIS IV detector. Specifically, two images for each crystal form were collected at 2θ = 0 and 90° at a temperature of 120 K with an exposure time of 6 min, detector distance ranging from 200 to 250 mm, and an oscillation angle ranging from 0.5 to 0.2° depending on the diffraction quality. Indexing was performed with iMosflm^71^ version 7.2.2 from the CCP4^72^ package. The unit cell parameters were automatically chosen by iMosflm.

### Activity Analysis of Single Crystals

Crystals of SDA_ec_, in either form, were harvested from four separate drops. Wash solution (10 μL of assay buffer) was first added to each drop and then the crystals were transferred to a 200 μL solution of assay buffer. Single crystals from the 200 μL drop were transferred to a seeding tool where the crystals were crushed to generate a slurry. The slurry was brought into an anaerobic glovebox, where the activity measurements were conducted. A total of six alternating reactions (150 μL) with and without the additional subunits and Fe^2+^ were performed by mixing 20 μL of crystal slurry, additional subunits (3 μM), Fe^2+^ (10 μM), and d,l-DTT (4 mM) together and incubating at 37 °C for 15 min. The reactions were initiated by adding l-cysteine to a final concentration of 1 mM. A quench solution of 37.5 μL of a 1:1 mixture of 20 mM *N*,*N*-dimethyl-*p*-phenylenediamine in 7.2 N HCl and 30 mM FeCl_3_ in 1.2 N HCl was added to the sample after 10 min. Sulfide concentration was determined as described above. Two independent triplicate runs were conducted, totaling six measurements for each sample.

## Native Mass Spectrometry Experiments

Native mass spectrometry (Native MS) was performed on two instruments for different purposes: an Exactive Plus with extended mass range (EMR) Orbitrap MS (Thermo Fisher Scientific, San Jose, CA) for high-resolution measurements or a Synapt G2 instrument (Waters Corporation, U.K.) equipped with an 8k RF generator for ion mobility measurements. Gold-coated tips prepared using a Sutter 1000 were used for nanoelectrospray ionization experiments.^73^ Fresh protein samples, including SDA_ec_, ISCU2, FXN, IscS, and IscU, were buffer exchanged into 200 mM ammonium acetate (pH = 8.5) using Micro Bio-Spin 6 Columns (Bio-Rad). Experimental and expected masses can be found in Table S8. All calculated masses excluded the N-terminal methionine (if present in the sequence). The calculated masses of SDA_ec_ and SDA_ec_ complexes included the mass of the covalently attached PLP and the assumed mass of the acyl-4′PPT ACP_ec_ was 523 Da. Masses of SDA_ec_/SDA_ec_U/SDA_ec_UF/ISCU2/FXN were measured under native conditions (200 mM ammonium acetate, pH = 8.5). Masses of SDA_ec_ subunits were also measured under denaturing conditions (1% formic acid). All masses were measured using the EMR.

### Protomer Exchange Experiments Using Native Mass Spectrometry

Protomer exchange experiments were performed on an EMR Orbitrap MS. The high resolution of EMR gives resolved peaks between subunit mixtures for quantification purposes. Instrument parameters were tuned to minimize collisional activation while retaining reasonable signal-to-noise. The mass spectrometer parameters used were set as *m*/*z* range 3000–10,000, capillary temperature 200–300 °C, S-Lens RF level 200, source DC offset 25 V, injection flatapole DC 16 V, inter flatapole lens DC 12 V, bent flatapole DC 7–12 V, transfer multipole DC offset 7–10 V, C-trap entrance lens tune offset 0 V, trapping gas pressure setting 7, in-source dissociation voltage 0 eV, HCD collision energy 10 eV, FT resolution 8750–35,000, positive ion mode, and ion maximum injection time 50–200 ms. For SDA_ec_ exchange experiments, a 1:1 ratio of ^15^N-SDA_ec_ and ^14^N-SDA_ec_ were mixed to initiate the exchange reaction. For subunit exchange of SDA_ec_U, ^15^N-SDA_ec_ and ^14^N-SDA_ec_ were incubated with ISCU2 distinctly using a 1:3 ratio for 30 min to form ^15^N-SDA_ec_U and ^14^N-SDA_ec_U complexes (α_2_β_2_γ_2_δ_2_). These complexes were mixed in a 1:1 ratio to initiate the exchange reaction. For the exchange of SDA_ec_UF, ^15^N-SDA_ec_ and ^14^N-SDA_ec_ were incubated with ISCU2 and FXN distinctly using a 1:3:3 ratio for 30 min to form ^15^N-SDA_ec_UF and ^14^N-SDA_ec_UF complexes (α_2_β_2_γ_2_δ_2_ε_2_). ^14^N-SDA_ec_UF and ^15^N-SDA_ec_UF were mixed in a 1:1 ratio to initiate the exchange reactions. The exchange of tagged IscS and untagged IscS was also investigated using a 1:1 ratio. For subunit exchange of IscS–IscU, tagged IscS and untagged IscS were incubated with IscU distinctly using a 1:3 ratio for 30 min to form untagged IscS–IscU and tagged IscS–IscU complexes (α_2_β_2_). The exchange reaction was initiated by mixing untagged IscS–IscU and tagged IscS–IscU complexes at a 1:1 ratio. At various time points, aliquots (4 μL) were taken for native MS analysis. Each spectrum was taken for 20 s. The initial MS data were collected using the Thermo Exactive software under the RAW format. The protein species were deconvoluted using the software program UniDec.^74^ All the exchange experiments were performed at room temperature.

### Cation Exchange Column Separation of SDA_ec_ Species

The native and variant SDA_ec_ samples were thawed rapidly and diluted to 60 μM with 50 mM HEPES, 250 mM NaCl, 10% glycerol, pH 7.5. The samples were diluted in half with cation buffer A (50 mM HEPES, 20 mM NaCl, 2% glycerol, pH 8.0) to a final concentration of 30 μM. Samples (1 mL) were injected onto a Mono S 5/50 GL (GE Healthcare) column using either a BioRad Quest or an AKTA FPLC and eluted using a step gradient of cation buffer B (50 mM Hepes, 1 M NaCl, 2% glycerol, pH 8) with steps at 15%, 30%, and 100%. For equilibrium experiments, the peak selected for isolation was concentrated to ∼400–500 μL using a 100 kDa cutoff Vivaspin 500 (GE Healthcare) by centrifugation at 10,000 RCF. The remaining sample was diluted to 1 mL with cation buffer A and reinjected and eluted using the same procedure. All experiments were performed at room temperature.

### Ion-Mobility Mass Spectrometry of SDAec/SDAecU/SDAecUF

Native ion-mobility mass spectrometry (IM-MS) was performed on a Synapt G2 instrument. Instrument parameters were tuned to maximize ion intensity but simultaneously preserve the native-like state of proteins as determined by IM. The instrument was set to a capillary voltage of 1–1.5 kV, source temperature of 30 °C, sampling cone voltage of 10 V, extraction cone voltage of 1 V, trap and transfer collision energy off, and backing pressure (5 mbar), trap flow rate at 8 mL/min, He cell flow rate at 200 mL/min, IMS flow rate at 50 mL/min. The T-wave settings for trap (310 ms^–1^/6.0 V), IMS (250 ms^–1^/9–12 V) and transfer (65 ms^–1^/2 V), and trap bias (25.0 V). MassLynx 4.1 (Waters) and Pulsar were used to deconvolute all recorded mass spectra.^75^ A sodium iodide solution was used to externally calibrate mass spectra. Experimental collisional cross-section (CCS) of ^14^N tagged SDA_ec_ (134.2 kDa), ^14^N untagged SDA_ec_ (129.3 kDa), SDA_ec_U (using ^14^N tagged SDA_ec_, 164.9 kDa), SDA_ec_UF (using ^14^N tagged SDA_ec_, 193.3 kDa) were determined following a well-documented protocol and a CCS database.^76,77^ Calibration curves (*R*^2^ > 0.978) were generated by using solutions of transthyretin (55.6 kDa), concanavalin A (103.0 kDa), and pyruvate kinase (237 kDa). Parameters for calculating the CCS using the online projected superposition approximation (PSA) Web server (psa.chem.fsu.edu) were set as follows: buffer gas of nitrogen, a temperature of 298 K, projection accuracy of 0.01, projection integration accuracy as 0.009, shape accuracy as 0.01, shape maxiter as 25, and shape mesh factor as 1.^48,50,51^ The models used for calculating the CCS were generated as described above.

### Additional Software and Figure Generation

Plots were generated in Excel (Microsoft) or KaliedaGraph (Synergy Software). Structural figures were generated using Chimera 1.11.2^78^ or PyMOL 2.4.^79^ High-resolution artboards and figures were developed using Inkscape (https://inkscape.org/) and GIMP (https://www.gimp.org/).

## Supporting Information Available

We provide tables for the SAXS data collection parameters and analyses of the AI-prepared SDA_ec_ complex, unit cell parameters for AI and TB-prepared SDA_ec_ samples, kinetic exchange of cysteine desulfurase protomers monitored by native mass spectrometry, collisional cross-section analysis of the ion mobility data and architectural summaries for eukaryotic cysteine desulfurase complexes, and calculated and measured masses for SDA_ec_ species. We also included figures for comparing the ready SDA_ec_ architecture with IscS, Michaelis–Menten kinetic results for the AI and TB-prepared SDA_ec_ complex and the SHQ variant, SAXS scattering curves of the SDA_ec_ complex, crystal images of the SDA_ec_ complex in the open and closed forms, native MS spectra of protomer exchange for the SDA_ec_ complex, results showing redistribution of SDA_ec_ species after cation exchange separation, images showing the rationale for the engineering of the SDA_ec_ complex to favor the ready form, IM-MS analysis of tagged vs untagged SDA_ec_ complex, and images comparing the active site region and FXN binding to the open, closed, and ready forms (PDF)
