# Supplementary material for: Frataxin Traps Low Abundance Quaternary Structure to Stimulate Human Fe–S Cluster Biosynthesis
Source: Biochemistry. 2025 Feb 5;64(4):903–16. doi: 10.1021/acs.biochem.4c00733 (PMC11840927; doi:10.1021/acs.biochem.4c00733)
Supplement: Supplementary file 1 — bi4c00733_si_001.pdf [file bi4c00733_si_001.pdf]

Supporting Information

Frataxin Traps Low Abundance Quaternary Structure to Stimulate Human Fe-S Cluster Biosynthesis

Seth A. Cory[†,a], Cheng-Wei Lin[†,a], Shachin Patra[a], Steven M. Havens[a], Christopher D. Putnam[b], Mehdi Shirzadeh[a], David H. Russell[a] and David P. Barondeau*[,a]

[a]Department of Chemistry, Texas A&M University, College Station, TX 77842, USA.

[b]Department of Medicine, University of California School of Medicine, La Jolla, CA, 92093-0660, USA.

Running Title: Frataxin Architectural Switch Mechanism

[†]Contributed equally to the manuscript

Table S1. SAXS parameters for the AI-prepared SDA_{ec} under high salt conditions compared to previous data.

	†SDA_{ec}	SDA_{ec}	SDA_{ec}	SDA_{ec}	SDA_{ec}
Data source	This manuscript	This manuscript	This manuscript	This manuscript	Markley (42)
Sample concentration (mg/mL)	1	2	4	Merged	N/A
Exposure time (sec)	1.5	1.5	0.6	0	N/A
Merged regions				1:(0.0109 - 0.07)<br>2:(0.1001 - 0.3202)	
Guinier analysis:					
I(0)	31.4 ± 0.2	82.1 ± 0.2	194.0 ± 0.4	2	904.6 ± 8.9
q range ($Å^{-1}$)	0.015-0.035	0.018 - 0.034	0.014 - 0.033	0.015-0.035	0.017 - 0.036
R_g (Å)	36.88 ± 0.25	37.71 ± 0.13	39.69 ± 0.12	36.88 ± 0.25	36.27 ± 0.47
$q R_g$ range	0.565 - 1.285	0.662 - 1.293	0.542 - 1.294	0.565 - 1.285	0.619 - 1.290
R^2	0.993	0.998	0.999	0.993	0.995
P(r) analysis:					
I(0)	31.6 ± 0.2	82.3 ± 0.2	194.5 ± 0.4	80.8 ± 0.32	923.9 ± 7.6
R_g (Å)	37.50 ± 0.22	38.17 ± 0.12	40.56 ± 0.12	37.07 ± 0.16	37.63 ± 0.38
d_{max} (Å)	127	129	144	128	124
q range ($Å^{-1}$)	0.0153 - 0.2171	0.0176-0.2121	0.0137-0.2015	0.0153 - 0.2171	0.0171 - 0.2200
χ^2 (total estimate from GNOM)	1.12	1.08	1.15	0.99	0.99
Mw analysis (Vc method, kDa)‡	134	129	139	126	139

†High ionic strength (100 mM sodium phosphate, 500 mM NaCl, 2% glycerol, 2 mM TCEP, pH = 8.0) data
N/A = not available
‡The high-angle data was truncated to 0.320 $Å^{-1}$ to maintain a consistent integration area.

*To whom correspondence should be addressed: Department of Chemistry, Texas A&M University, College Station, TX 77842, USA. Telephone: 979-458-0735. E-mail: barondeau@tamu.edu

Table S2. SAXS parameters for the AI-prepared SDA_{ec} under low salt conditions compared to previous data.

	†SDA_{ec}	SDA_{ec}	SDA_{ec}	SDA_{ec}	SDA_{ec}	SDA_{ec}
Data source	This manuscript	This manuscript	This manuscript	Cygler/Lill (15)	Cygler/Lill (15)	Cygler/Lill (15)
Sample concentration (mg/mL)	1.0	2.0	4.0	1.32	2.65	5.29
Guinier analysis:						
q range ($Å^{-1}$)	0.013-0.032	0.014 - 0.028	0.014 - 0.025	0.014 - 0.030	0.012 - 0.028	0.017 - 0.026
R_g (Å)	39.89 ± 0.24	44.86 ± 0.18	51.48 ± 0.13	42.99 ± 0.29	45.65 ± 0.13	50.72 ± 0.18
qR_g range	0.52 - 1.28	0.62 - 1.24	0.73 - 1.28	0.62 - 1.30	0.57 - 1.30	0.87 - 1.30
R^2	0.997	0.998	0.999	0.996	0.999	0.999
P(r) analysis:						
R_g (Å)	40.31 ± 0.19	45.54 ± 0.12	53.30 ± 0.15	44.18 ± 0.27	46.56 ± 0.18	52.15 ± 0.15
d_{max} (Å)	132	154	189	142	158	177
q range ($Å^{-1}$)	0.014 - 0.200	0.014-0.178	0.014-0.155	0.0143 - 0.1863	0.0124 - 0.1754	0.0171 - 0.1575
χ^2 (total estimate from GNOM)	1.04	1.39	1.48	0.90	0.76	1.01
Mw analysis (Vc method, kDa)‡	167	178	220	180	189	233

† Low ionic strength (50 mM Hepes, 250 mM NaCl, 2% glycerol, 2 mM TCEP, pH = 7.5) data

‡ The high-angle data was truncated to 0.320 $Å^{-1}$ to maintain a consistent integration area.

Table S3. Indexing of open (space group $P2_12_12_1$) and closed (space group $H3_2$) crystal forms from AI and TB prepared SDA_{ec} samples.

		Unit Cell Parameters					
**Crystal Form**	**Preparation**	**a (Å)**	**b (Å)**	**c (Å)**	**α (°)**	**β (°)**	**γ (°)**
Open (5USR)*	AI-method	125.5	147.8	168.5	90	90	90
Open	AI-method	125.8	147.9	168.1	90	90	90
Open (-allylglycine)	AI-method	122.1	144.1	163.6	90	90	90
Open	TB-method	125.2	146.8	166.7	90	90	90
Closed (5WGB)*	TB-method	140.8	140.8	203.3	90	90	120
Closed	TB-method	139.8	139.8	200.6	90	90	120
Closed	AI-method	139.2	139.2	200.5	90	90	120

*Published unit cell parameters.

Table S4. Kinetic exchange of cysteine desulfurase protomers monitored by native mass spectrometry.

**Time**	**2 min**	**6 min**	**10 min**	**15 min**	**23 min**
tagged SDA_{ec}	‡0.070 ± 0.008	0.149 ± 0.016	0.153 ± 0.013	0.228 ± 0.028	0.404 ± 0.033
tagged $SDA_{ec}U$	0.000	0.000	0.000	0.000	0.000
tagged $SDA_{ec}UF$	0.000	0.000	0.000	0.000	0.000
untagged SDA_{ec}	0.000	0.000	0.000	0.004 ± 0.004	0.003 ± 0.005
untagged $SDA_{ec}U$	0.000	N/A	N/A	N/A	N/A
untagged $SDA_{ec}UF$	0.000	N/A	N/A	N/A	N/A
IscS	0.016 ± 0.000	N/A	N/A	N/A	N/A
IscS-IscU	0.000	N/A	N/A	N/A	N/A
**Time**	**35 min**	**55 min**	**80 min**	**120 min**	**24 hr**
tagged SDA_{ec}	0.448 ± 0.028	0.640 ± 0.034	0.713 ± 0.052	0.833 ± 0.041	N/A
tagged $SDA_{ec}U$	0.000	0.000	0.000	0.000	N/A
tagged $SDA_{ec}UF$	0.000	0.000	0.000	0.000	N/A
untagged SDA_{ec}	0.011 ± 0.008	0.015 ± 0.011	0.025 ± 0.009	0.039 ± 0.007	0.787 ± 0.060
untagged $SDA_{ec}U$	0.000	N/A	0.000	0.000	N/A
untagged $SDA_{ec}UF$	0.000	N/A	0.000	0.000	N/A
IscS	0.078 ± 0.012	0.141 ± 0.02	N/A	0.311 ± 0.007	N/A
IscS-IscU	0.000	0.000	N/A	0.000	N/A

‡The values in the table are shown as the amount of exchanged dimer ($^{15}N^{14}N$) divided by unexchanged dimer ($^{14}N^{14}N$ and $^{15}N^{15}N$) or as the amount of exchanged IscS divided by the unexchanged dimer (tagged and untagged). N/A is not available.

Table S5. Collision cross section (Ω) of tagged SDA_{ec} species.

Sample	Mass (kDa)	z	Form	Ω_{Exp} (Å^2)	Ω_{PA} (Å^2)	Ω_{PSA} (Å^2)
SDA_{ec}	134.2	23	compact*	6928 ± 108	Ready = 7076	Ready = 7721 ± 51
			extended	7203 ± 160	Closed = 6944	Closed = 7692 ± 48
		24	compact*	7021 ± 130	Open = 7287	Open = 8031 ± 39
			extended	7267 ± 111		
$S^{SHQ}DA_{ec}$	134.2	23	compact	7048 ± 20		
			extended	7303 ± 29		
		24	compact	7145 ± 18		
			extended	7389 ± 41		
$SDA_{ec}U$	164.9	26	compact*	8194 ± 140	Closed = 8099	Closed = 8854 ± 44
			extended	8737 ± 48	Ready = 8126	Ready = 8829 ± 51
		27	compact*	8390 ± 81	Open = 8211	Open = 9251 ± 48
			extended	8802 ± 21		
$S^{SHQ}DA_{ec}U$	164.9	26	compact	8108 ± 20		
			extended	8693 ± 20		
		27	compact	8179 ± 19		
			extended	8827 ± 33		
$SDA_{ec}F$	164.1	26	compact	8041 ± 23	Ready = 7868	Ready = 8964 ± 44
		27	compact	8051 ± 37		
$S^{SHQ}DA_{ec}F$	164.1	26	compact	8107 ± 24		
		27	compact	8107 ± 24		
$SDA_{ec}UF$	193.3	27	compact	9333 ± 22	Ready = 8713	Ready = 9758 ± 75
		28	compact	9370 ± 52		
$S^{SHQ}DA_{ec}UF$	193.3	27	compact	9279 ± 25		
		28	compact	9418 ± 30		

*Calculated using batch 3 of SDA_{ec} showing in Fig. S10

Table S6. Architectural summary for human cysteine desulfurase complexes.

	Structure(s) determined	Cation exchange chromatography	IM-MS	Activity (relative to $SDA_{ec}UF$)[14]	Tentative solution architecture(s)
**SDA_{ec}**	Open and closed	Mainly peak 3, partially peak 2 (<10%)	Mainly extended, partially compact (<30%)	~6%	Mainly open, partially closed or ready (<30%)
**$SDA_{ec}U$**	Ready	ND	Mainly extended, partially compact (<30%)	~6%	Mainly open, partially closed or ready (<30%)
**$SDA_{ec}UF$**	Ready	ND	Compact	100%	Ready
**SDA_{ec} SHQ**	ND	Mainly peak 3, enhanced peak 2 (~20%)	Mainly extended, enhanced compact (~50%)	~20%	Mainly open, enhanced ready (~20-50%)
**$S^{SHQ}DA_{ec}U$**	ND	ND	Mainly extended, enhanced compact (~50%)	ND	Mainly open, enhanced ready (~20-50%)
**$S^{SHQ}DA_{ec}UF$**	ND	ND	Compact	~80%	Ready

Table S7. Small-angle X-ray scattering data collection parameters.

	SDA_{ec}
Organism	*H. sapiens* , *E* . coli
Source	Recombinant
Source of Data	This work
Theoretical Mw, excluding cofactors (kDa)	133 kDa
Sample concentrations (mg/mL)	1, 2, 4
Sample buffer	100 mM phosphate<br>500 mM NaCl<br>2% glycerol<br>2 mM TCEP<br>pH = 8.0
Instrument/data processing	SIBYLS ALS 12.3.1
q range ($Å^{-1}$)	0.011-0.565
Wavelength (Å)	1.27
Cell thickness (mm)	1.5
Sample to detector distance (m)	1.5
Temperature (^{o}C)	10
Exposure time (sec)	10
Delta/time slicing	Every 0.3 seconds
Total frames	32

Table S8. Calculated and measured masses for SDA_{ec} species.

Species		Calculated Mass (Da)	Measured Mass (Da)	Measured Mass range
^{14}N-untagged SDA_{ec}		129097	129265 ± 64	129201−129329
^{15}N-untagged SDA_{ec}		130693	130838 ± 70	130768−130908
^{14}N-untagged $SDA_{ec}U$		159521	159708 ± 99	159609−159807
^{15}N-untagged $SDA_{ec}U$		161117	161285 ± 130	161155−161415
^{14}N-untagged $SDA_{ec}UF$		187997	188175 ± 97	188078−188272
^{15}N-untagged $SDA_{ec}UF$		189593	189770 ± 124	189646−189894
^{14}N-tagged SDA_{ec}		134135	134235 ± 76	134159−134311
^{15}N-tagged SDA_{ec}		135809	135892 ± 72	135820−135964
^{14}N-tagged $SDA_{ec}U$		164559	164880 ± 193	164687−165073
^{15}N-tagged $SDA_{ec}U$		166233	166596 ± 181	166415−166777
^{14}N-tagged $SDA_{ec}UF$		193035	193329 ± 212	193117−193541
^{15}N-tagged $SDA_{ec}UF$		194709	195004 ± 155	194849−195159
SDA_{ec}_SHQ		134149	134259 ± 69	15203−15219
ISCU2-Zn		15212	15211 ± 8	14231−14245
FXN		14238	14238 ± 7	134190−134328
**Species**	**Subunit**			
^{14}N-untagged SDA_{ec}	S	44717	44716 ± 17	44700−44734
	D	10611	10610 ± 12	10599−10623
^{15}N-untagged SDA_{ec}	S	45273	45262 ± 18	45255−45291
	D	10760	10757 ± 11	10749−10771
^{14}N-tagged SDA_{ec}	S	47197	47194 ± 18	47179−47215
	D	10611	10610 ± 12	10599−10623
^{15}N-tagged SDA_{ec}	S	47792	47785 ± 18	47774−47810
	D	10760	10758 ± 11	10749−10771
SDA_{ec}_SHQ	S	47211	47208 ± 28	47183−47239
	D	10611	10611 ± 13	10598−10624

*Errors of masses are estimated from full width at half maximum.

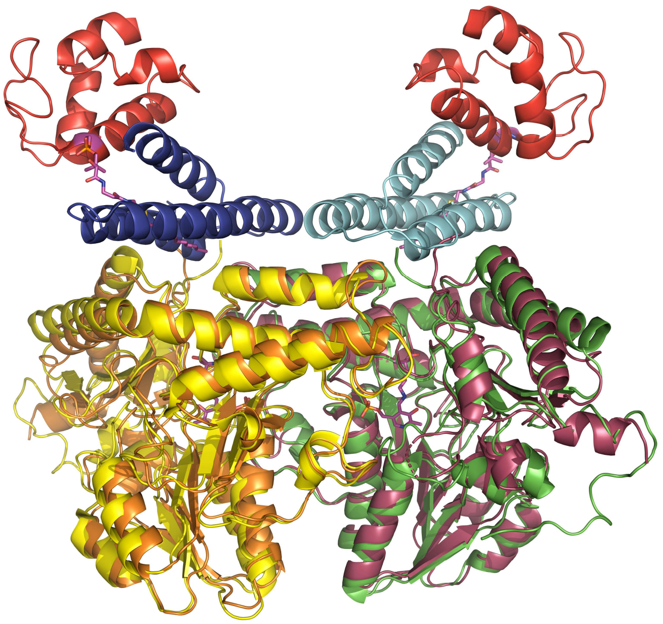

Figure S1. Comparison of the ready SDA$_{ec}$ architecture with IscS. Structure of the SDA$_{ec}$ in the ready form (oriented as in Figure 1C; NFS1 colored in green and yellow, ISD11 in blue and cyan, and ACP$_{ec}$ in red) overlaid with IscS (subunits in orange and purple). Protein cofactors shown in magenta.

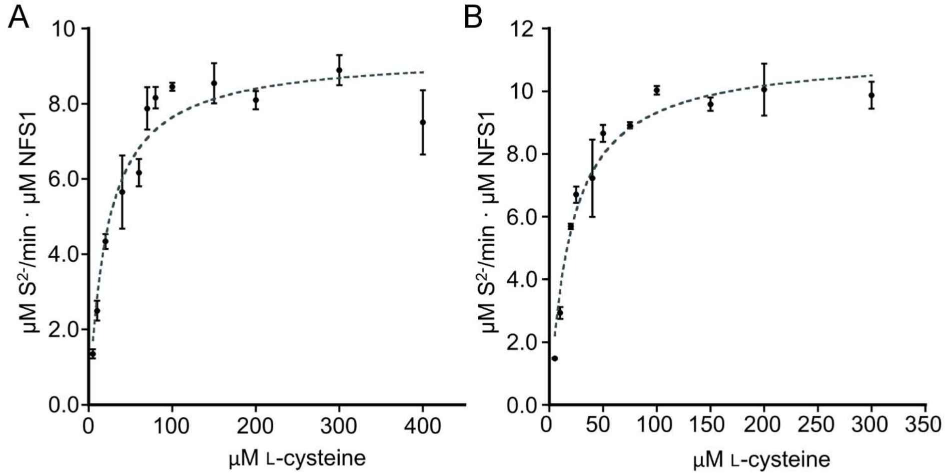

Figure S2. SDA$_{ec}$ complexes prepared using different expression conditions exhibit similar kinetics for cysteine turnover. SDA$_{ec}$ (0.5 μM) samples prepared using the (**A**) AI and (**B**) TB conditions were assayed in the presence of ISCU2 (1.5 μM), FXN (1.5 μM), Fe^{2+} (5 μM) and D-L-DTT (4 mM). Error bars are replicate errors (n = 3). The dashed lines through the data are the fits to the Michaelis-Menten equation.

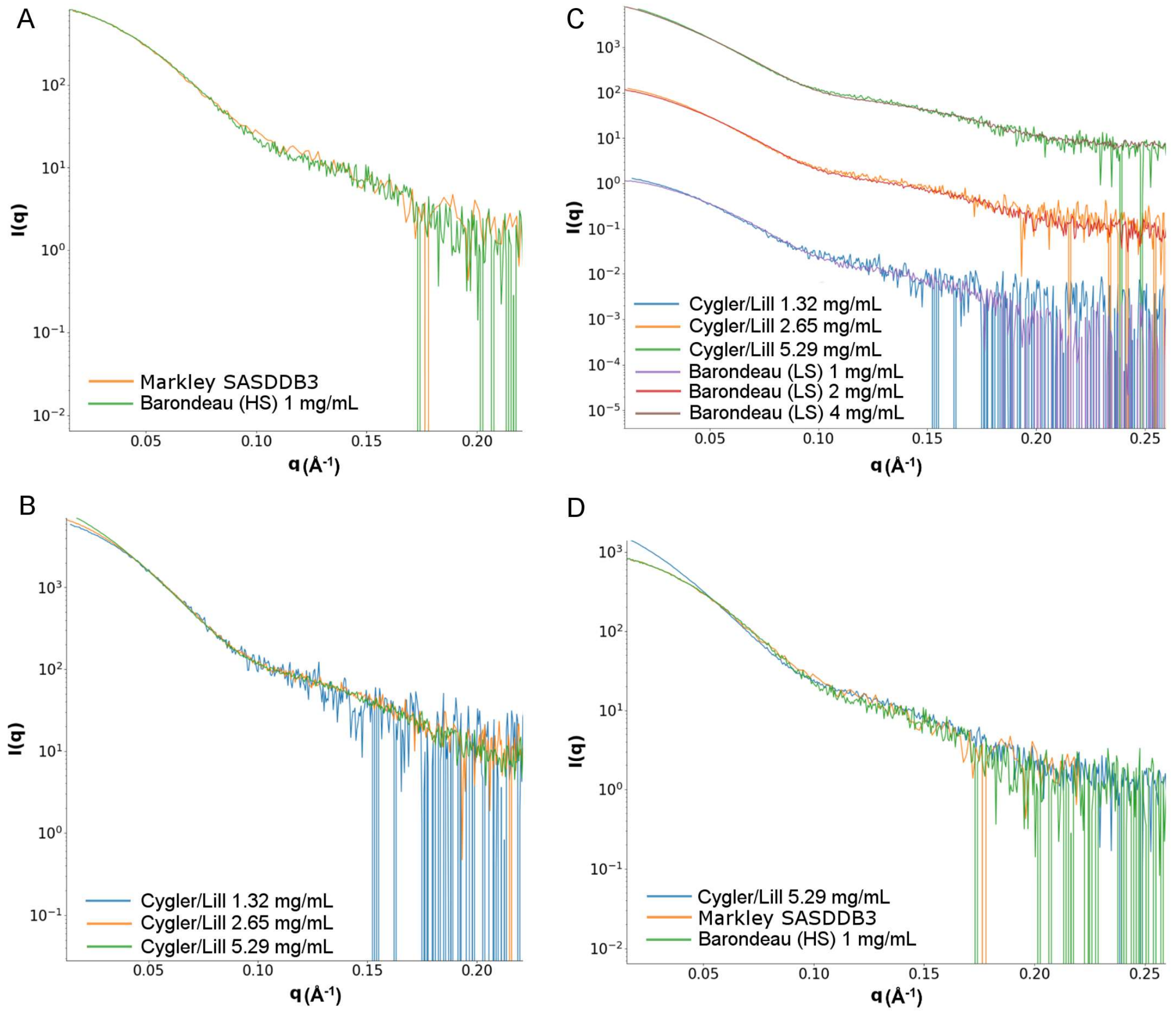

Figure S3. Comparison of scattering curves for AI-prepared SDA$_{ec}$ and previously published data. Intensities are plotted on a logarithmic scale. (**A**) Scattering curves for the AI-prepared SDA$_{ec}$ complex under high salt conditions (100 mM sodium phosphate, 500 mM NaCl, 2% glycerol, 2 mM TCEP, pH = 8.0) and the SASDDB3 data from the Markley group. (**B**) Scattering curves from the Cygler/Lill groups at different protein concentrations. The highest concentration sample appears to have been utilized in their manuscript. (**C**) Scattering curves from the AI-prepared SDA$_{ec}$ complex under low salt conditions (250 mM NaCl, 2% glycerol, 2 mM TCEP, pH = 7.5) compared to those from the Cygler/Lill groups. (**D**) Overlay of the scattering curve for the AI-prepared SDA$_{ec}$ sample under high ionic strength conditions with the scattering curves from the Cygler/Lill and Markley data.

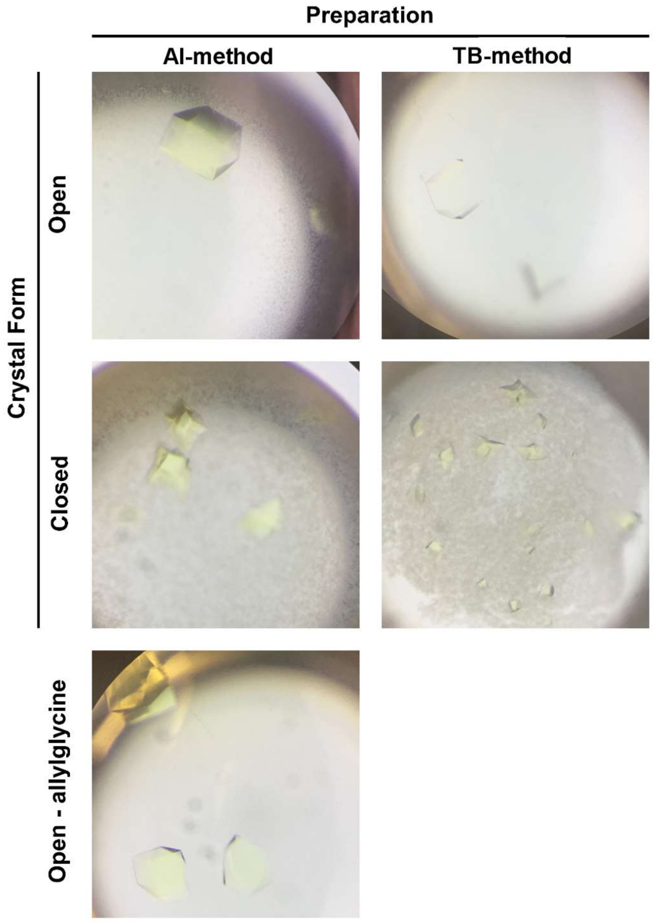

Figure S4. AI and TB prepared SDA_{ec} samples form crystals in both open and closed architectures. AI and TB prepared SDA_{ec} samples were crystallized using trials similar to the published conditions. The unit cell parameters were consistent with the open and closed forms (Table S3).

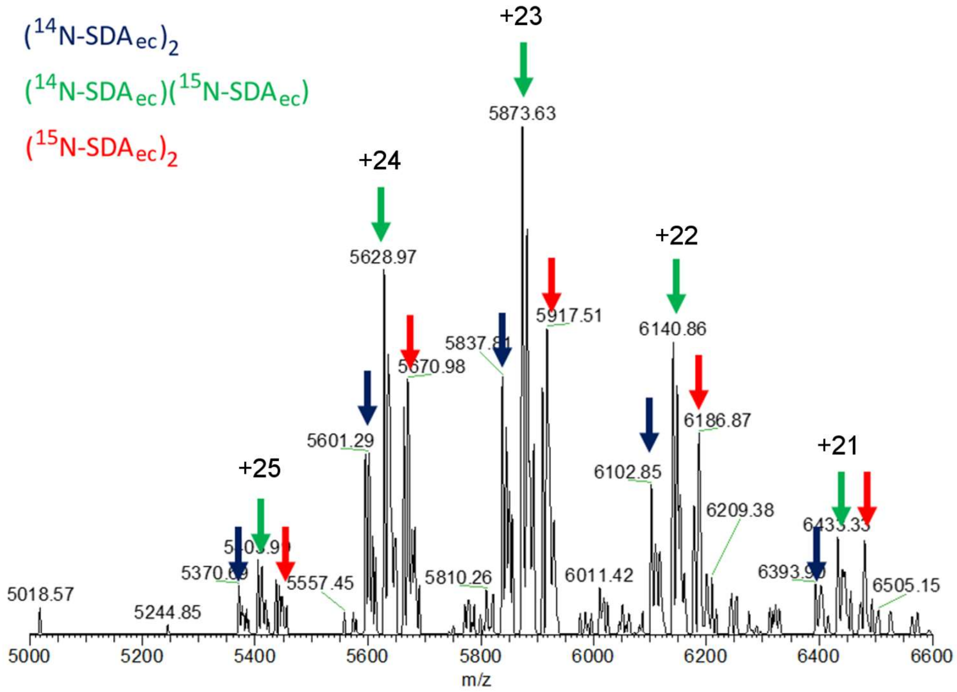

Figure S5. Native MS spectra showing protomer exchange for cysteine desulfurase complexes. Representative native MS spectra (2 hr time point from Fig 4) for the reaction of a 1:1 ratio of His-tagged $^{14}N\text{-}SDA_{ec}$ and $^{15}N\text{-}SDA_{ec}$ complexes to form a mixed structure containing ^{14}N and ^{15}N labeled protomers. The charge states +25 to +21 are shown for the three complexes.

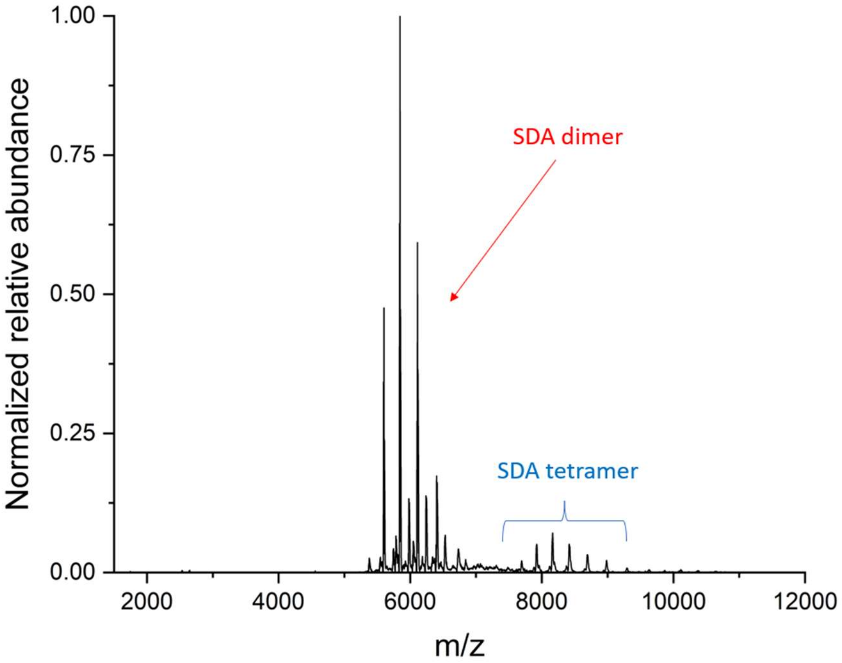

Figure S6. Native mass spectrometry analysis of the SDA$_{ec}$ complex. The native complex was primarily in the $\alpha_2\beta_2\gamma_2$ dimeric form with a small fraction of tetrameric species.

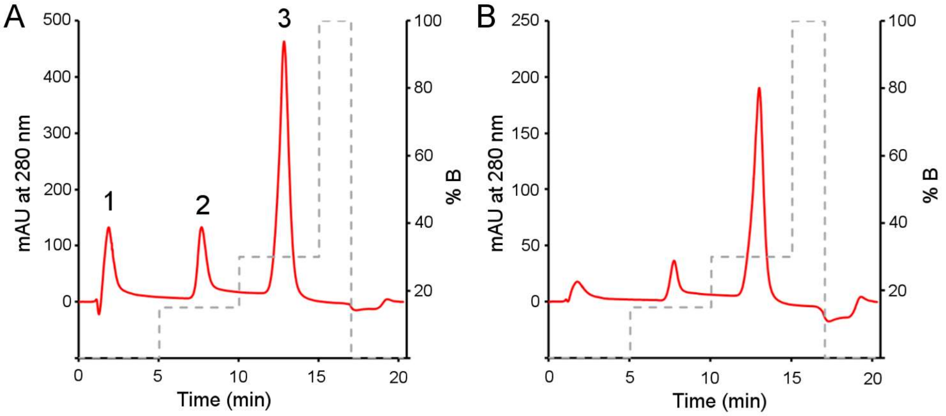

Figure S7. Redistribution of SDA_{ec} species revealed by cation exchange chromatography. The 280 nm traces of peaks eluting from a cation exchange column are shown in red. The gradient in buffer B concentration is displayed in grey dashed lines. (**A**) 30 μM injection of SDA_{ec} onto a Mono S 5/50 GL column and elution by step gradient identifies three species. (**B**) Peak 3 in panel A was isolated and concentrated to approximately 400 μL and diluted to 1 mL with cation buffer A prior to re-injection. All species identified in panel A are present in panel B, which suggests the sample underwent a re-equilibration process.

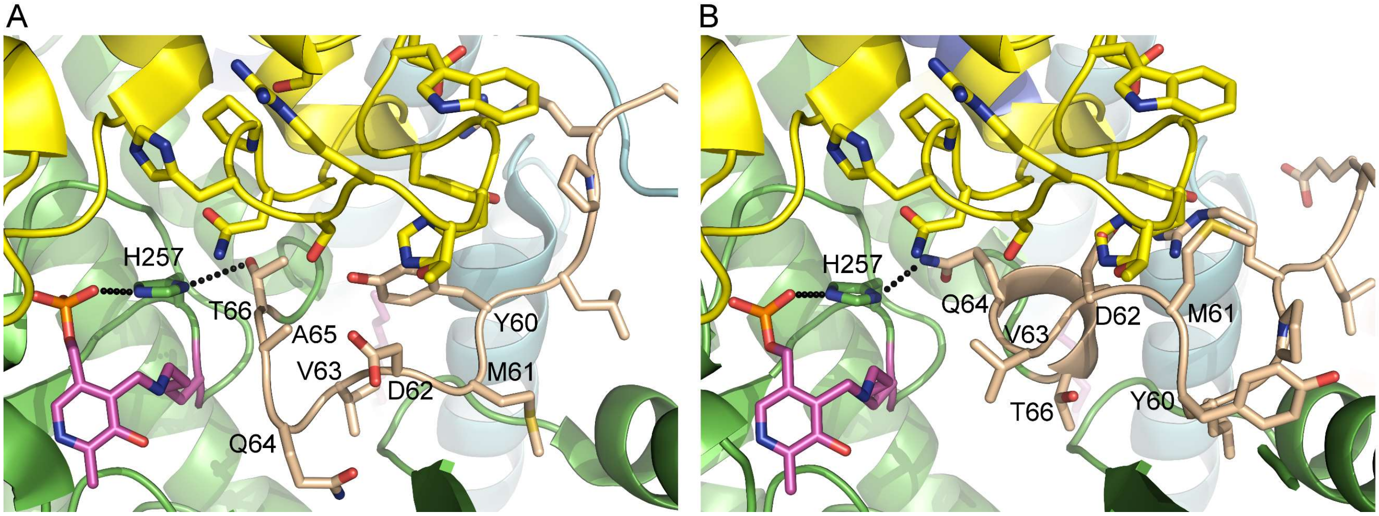

Figure S8. N-terminal engineering of SDA_{ec} to favor the ready architecture. (**A**) In the ready architecture, the NFS1 catalytic subunits (green and yellow) have similar protein interfaces to IscS (Fig. S1). T66 from the ready architecture forms a hydrogen bond to H257 that, in turn, hydrogen bonds to the PLP cofactor (magenta). (**B**) In the open architecture, Q64 hydrogen bonds to H257, which results in an N-terminal structural rearrangement compared to the ready form. Residues, especially M61 and D62, from this region of the green NFS1 subunits in the open form are shown in wheat and would have steric clashes with the second catalytic subunit from the ready form (docked into structure, shown in yellow). The Q64S substitution would disfavor the N-terminal rearrangement of the open form and, instead, promote the formation of the ready configuration.

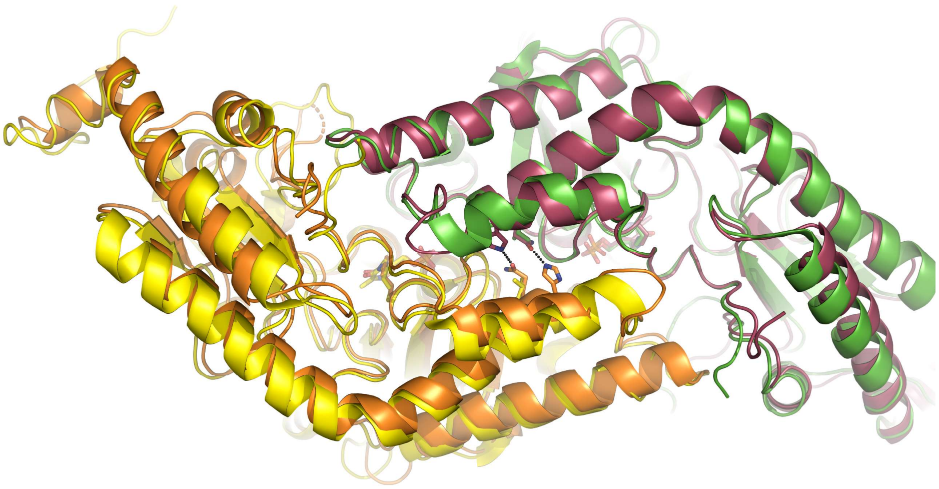

Figure S9. Substituting a hydrogen-bonding pair of residues from IscS to stabilize the ready SDA_{ec} architecture. In the IscS structure (subunits in orange and purple), the dimer interface is stabilized by two hydrogen bonds between residues H247 and Q248 of the two subunits. The equivalent residues in human NFS1 are P299 and L300, which form weaker Vander Waals interactions at the dimer interface. The NFS1 variant P299H L300Q is predicted to preferentially stabilize the ready architecture.

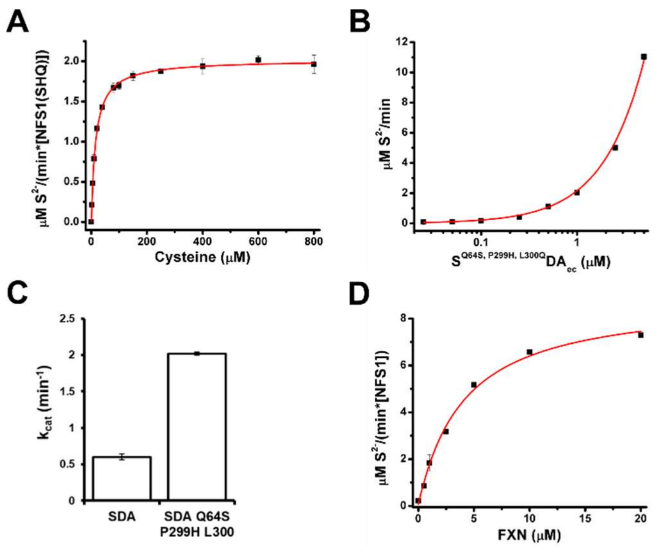

Figure S10. The SHQ variant has inherently higher activity than SDA$_{ec}$. (**A**) Michaelis-Menten kinetics of the S$^{Q64S\ P99H\ L300Q}$DA$_{ec}$ (SHQ) variant. (**B**) Variation of the concentration of SHQ while keeping the L-cysteine concentration at 1 mM leads to a linear increase (R^2 - 0.9977) of the activity with a slope of 2.16 ± 0.03, which is very close to the k_{cat} value (2.02 ± 0.02). (**C**) Comparison of k_{cat} of SDA$_{ec}$ and the SHQ variant. (**D**) FXN in the presence of ISCU2 activates the SHQ variant to the SDA$_{ec}$UF level but FXN binds weakly (K_D = 3.8 ± 0.6 μM for SHQ compared to 0.22 ± 0.05 μM for the native enzyme).

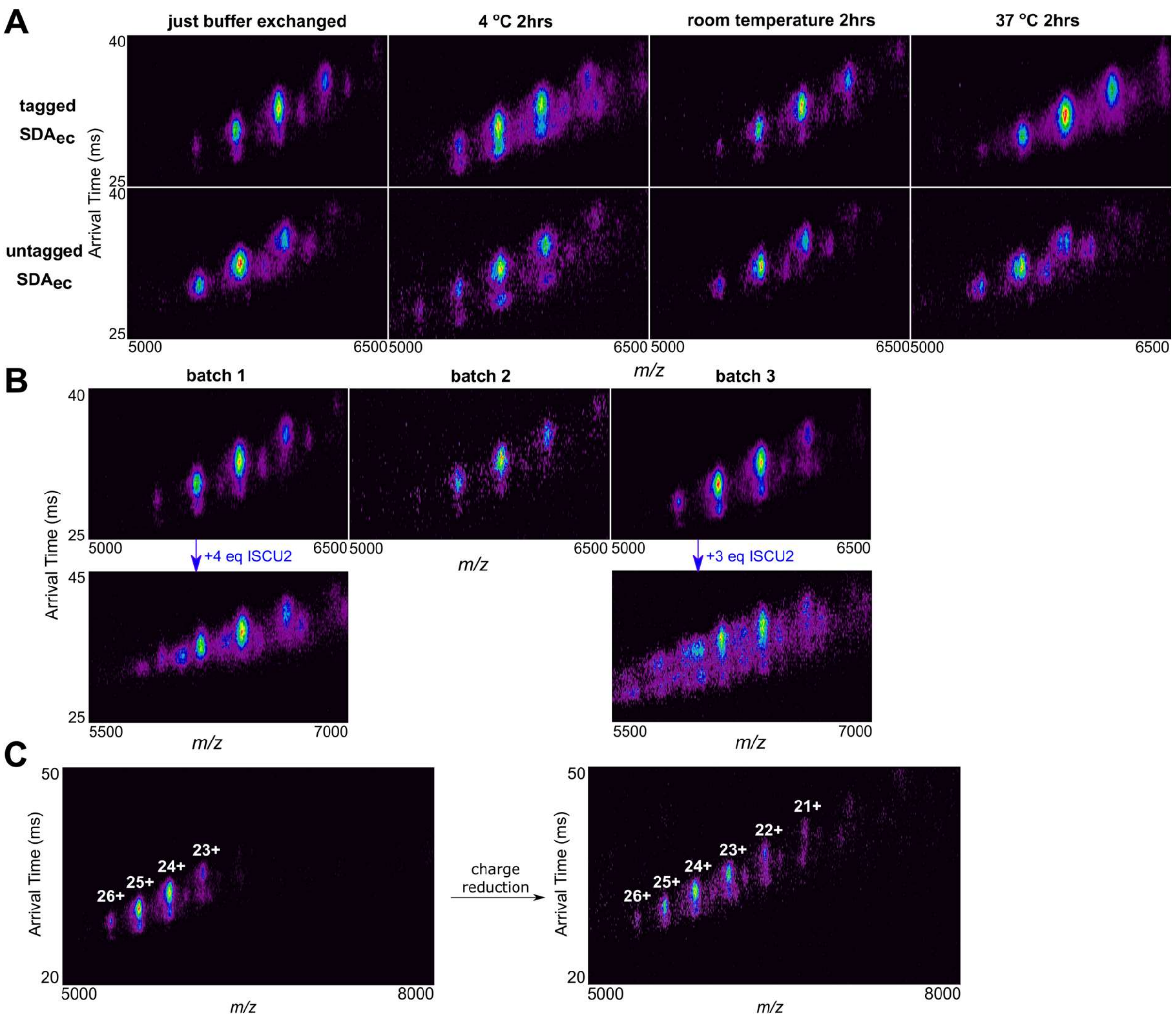

Figure S11. Distinct species in IM-MS spectra reflect different architectures in solution. (**A**) IM-MS analysis of untagged and tagged SDA_{ec} samples incubated in 200 mM ammonium acetate (pH = 8.5) at different temperatures. Both untagged and tagged SDA_{ec} were predominantly in the slower migrating form upon buffer exchange. When stored at 4 °C, both untagged and tagged SDA_{ec} showed an increase in the faster migrating form (~30% for tagged and ~39% for untagged, which were estimated using the total ion current of corresponding species extrapolated from the ion mobility spectra). When stored at room temperature or 37 °C, both tagged and untagged SDA_{ec} samples stayed predominantly in the slower migrating form. For data consistency, all IM-MS spectra in this study were measured within two-hours of incubating buffer exchanged samples at room temperature. (**B**) IM-MS analysis of different batches of tagged SDA_{ec}. Slight differences in the distribution of species were observed (faster migrating species ~17% for batch 1, ~10% for batch 2, ~26% for batch 3). IMS of batch 1 and batch 3 in the presence of ISCU2 are also shown. (**C**) IM-MS spectrum of tagged SDA_{ec} batch 3 in 200 mM ammonium acetate, pH = 8.5 (left panel) or 200 mM ammonium acetate, 20 mM triethylammonium acetate, pH = 8.5 (right panel) are shown. The charge reduced (high m/z) species in the right panel still retains two conformations, suggesting the slower migrating form is not a result of collisional activation.

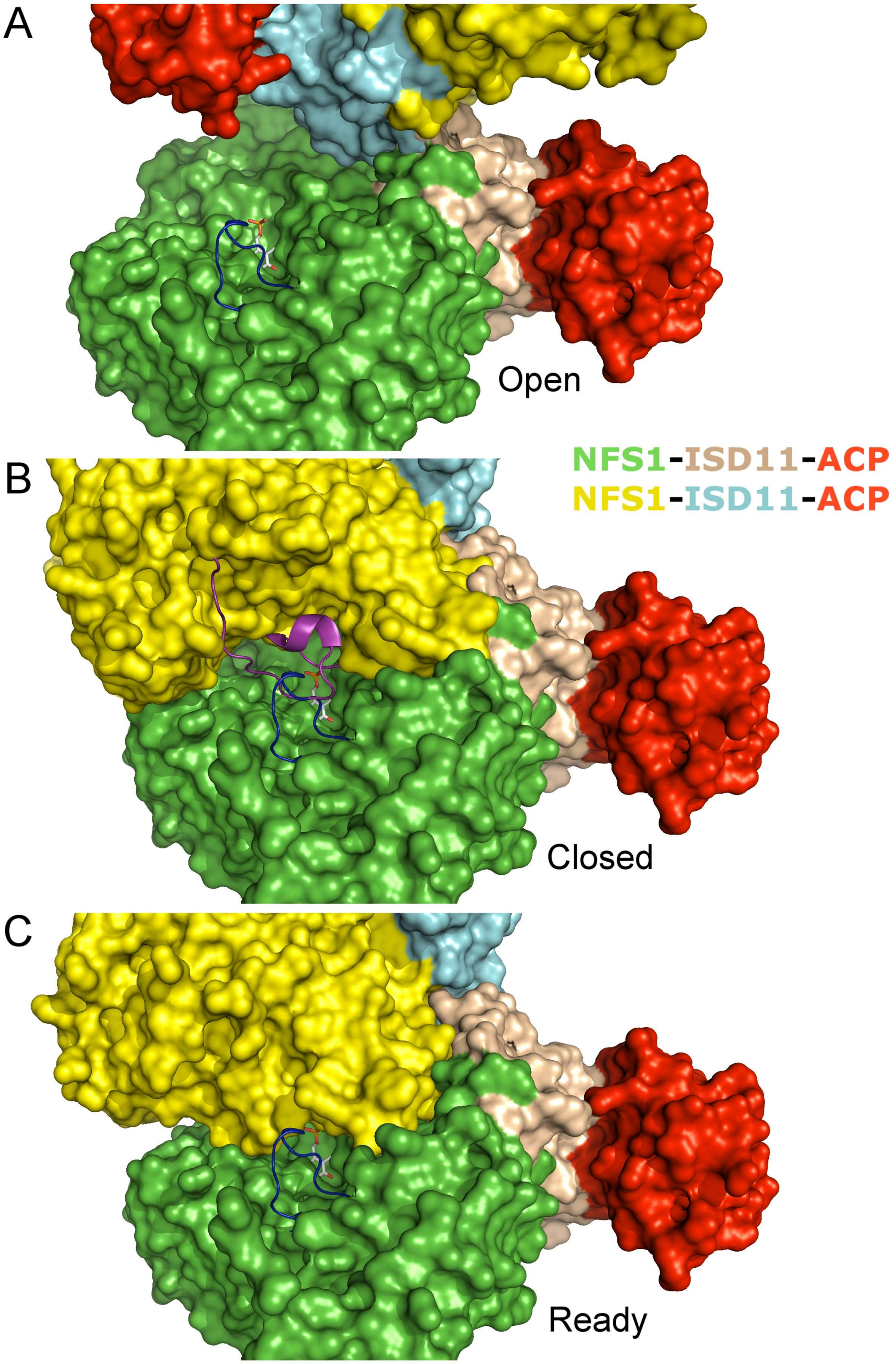

Figure S12. Differences in the active sites for the three SDA_{ec} architectures. (**A**) Surface representation of the open SDA_{ec} model highlighting the solvent exposed PLP (white) and the C381-containing mobile S-transfer loop (dark blue). (**B**) Surface representation of the closed SDA_{ec} model that also shows a structural element (purple) from the other subunit that may inhibit the function of the mobile S-transfer loop. Notably, the structural element and mobile loop are disordered in the closed crystal structure. (**C**) Surface representation of the ready SDA_{ec} model. The NFS1 subunits are colored in green and yellow, the ISD11 in cyan and wheat, and ACP in red.

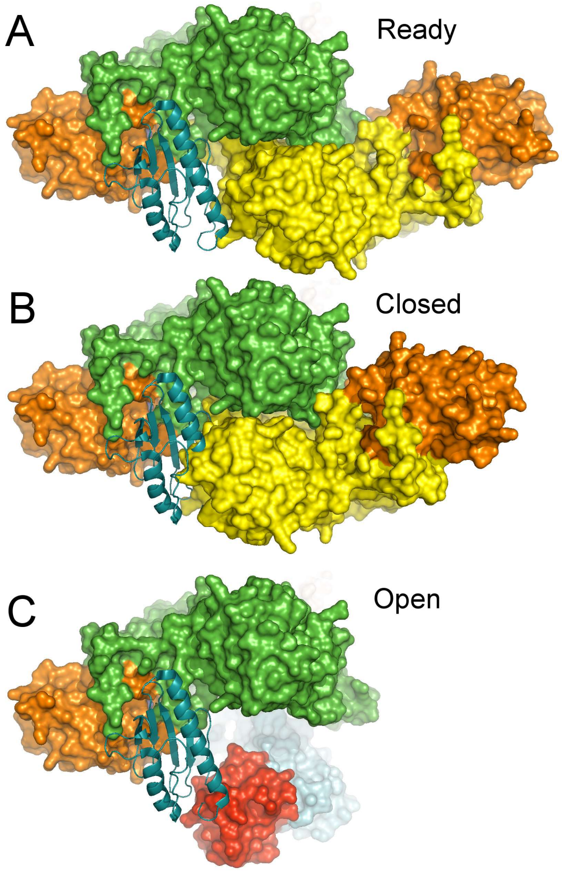

Figure S13. Comparison of FXN binding to the different architectural models. Structure of the $SDA_{ec}U$ complex with one FXN bound in the ready (**A**), closed (**B**), and open (**C**) forms. NFS1 surfaces colored in green and yellow, ISD11 in wheat and cyan, ACP_{ec} in red, ISCU2 in orange, and FXN as a teal ribbon diagram. The closed and open forms are based on the binding interaction in the $SDA_{ec}UF$ cryo-EM structure in the ready form. FXN binding has either significant steric clash (closed) or loses interactions (open) with the NFS1 subunit (yellow) from the other protomer.
